# Laboratory tools for the direct detection of bacterial respiratory infections and antimicrobial resistance: a scoping review

**DOI:** 10.1177/10406387241235968

**Published:** 2024-03-08

**Authors:** Olufunto O. Adewusi, Cheryl L. Waldner, Patrick C. Hanington, Janet E. Hill, Claire N. Freeman, Simon J. G. Otto

**Affiliations:** HEAT-AMR (Human-Environment-Animal Transdisciplinary Antimicrobial Resistance) Research Group, University of Alberta, Edmonton, AB, Canada; School of Public Health, University of Alberta, Edmonton, AB, Canada; Departments of Large Animal Clinical Sciences, Western College of Veterinary Medicine, University of Saskatchewan, Saskatoon, SK, Canada; School of Public Health, University of Alberta, Edmonton, AB, Canada; Veterinary Microbiology, Western College of Veterinary Medicine, University of Saskatchewan, Saskatoon, SK, Canada; Departments of Large Animal Clinical Sciences, Western College of Veterinary Medicine, University of Saskatchewan, Saskatoon, SK, Canada; HEAT-AMR (Human-Environment-Animal Transdisciplinary Antimicrobial Resistance) Research Group, University of Alberta, Edmonton, AB, Canada; Healthy Environments Thematic Area Lead, Centre for Healthy Communities, University of Alberta, Edmonton, AB, Canada; School of Public Health, University of Alberta, Edmonton, AB, Canada

**Keywords:** antimicrobial resistance, antimicrobial stewardship, laboratory testing, long-read metagenomic sequencing, respiratory disease

## Abstract

Rapid laboratory tests are urgently required to inform antimicrobial use in food animals. Our objective was to synthesize knowledge on the direct application of long-read metagenomic sequencing to respiratory samples to detect bacterial pathogens and antimicrobial resistance genes (ARGs) compared to PCR, loop-mediated isothermal amplification, and recombinase polymerase amplification. Our scoping review protocol followed the Joanna Briggs Institute and PRISMA Scoping Review reporting guidelines. Included studies reported on the direct application of these methods to respiratory samples from animals or humans to detect bacterial pathogens ±ARGs and included turnaround time (TAT) and analytical sensitivity. We excluded studies not reporting these or that were focused exclusively on bioinformatics. We identified 5,636 unique articles from 5 databases. Two-reviewer screening excluded 3,964, 788, and 784 articles at 3 levels, leaving 100 articles (19 animal and 81 human), of which only 7 studied long-read sequencing (only 1 in animals). Thirty-two studies investigated ARGs (only one in animals). Reported TATs ranged from minutes to 2 d; steps did not always include sample collection to results, and analytical sensitivity varied by study. Our review reveals a knowledge gap in research for the direct detection of bacterial respiratory pathogens and ARGs in animals using long-read metagenomic sequencing. There is an opportunity to harness the rapid development in this space to detect multiple pathogens and ARGs on a single sequencing run. Long-read metagenomic sequencing tools show potential to address the urgent need for research into rapid tests to support antimicrobial stewardship in food animal production.

Laboratory tests have provided life-saving information to detect, characterize, and inform management and treatment decisions for bacterial infections in human and animal health. The increased burden of antimicrobial-resistant bacterial infections in humans and animals,^12,36,56,124^ antimicrobial resistance (AMR) genes in the environment,^100,148^ and the potential for their transmission between humans and animals^80^ have increased pressure for food animal livestock production to demonstrate antimicrobial stewardship.^96^ In 2015, the World Health Organization (WHO) developed a Global Action Plan for AMR, urging every member state to adopt and adapt 5 objectives to their national context.^153^ The WHO, the Food and Agriculture Organization, and the World Organisation for Animal Health (formerly the OIE) made joint commitments to improve antimicrobial stewardship in the human and animal sectors, including investment in new laboratory tests to detect AMR.^49^ Laboratory testing is an important tool of evidence-based veterinary medicine, which promotes antimicrobial stewardship in livestock production.^49^

In countries such as Canada, upper and lower respiratory tract infections affect both adults and children and result in the administration of antimicrobials.^56^ Similarly, bacterial respiratory infections in animals, such as *Mesomycoplasma* (*Mycoplasma*) *hyopneumoniae* and *Actinobacillus pleuropneumoniae* in swine,^39^ multiple organisms in horses (e.g., *Actinobacillus suis, Streptococcus equi*, and *Streptococcus equi* subsp. *zooepidemicus*),^32^ and *Bordetella bronchiseptica* in dogs and cats^131^ can impact animal health and welfare and similarly result in antimicrobial use (AMU). Bovine respiratory disease (BRD) in feedlot cattle causes high morbidity and mortality and requires AMU for management.^18^ With complex pathogenesis involving many bacteria and viruses,^53,114^ diseases such as BRD require timely and accurate management and treatment decisions^20^ where the need for AMU should be informed by rapid laboratory tests in addition to known risk factors and clinical signs.^4,53,114^ Detection of AMR in bacteria can further target antimicrobial therapy by informing the choice of antimicrobials where required to manage disease, with point-of-care (POC) testing being the ultimate goal.^24,33,91,110^

Rapid and immediate laboratory techniques for POC identification of pathogens and AMR determinants to guide antimicrobial selection in individual animals are not commercially available. Long-read sequencing platforms such as Oxford Nanopore Technology (ONT)^116^ and Pacific Biosciences (PacBio) single-molecule real-time (SMRT) sequencing^87^ are of particular interest for AMR detection because they have the potential to provide more timely results through direct application of metagenomic sequencing to clinical samples. Short-read sequencing platforms ([NovaSeq, HiSeq, NextSeq, MiSeq; Illumina], [MGISEQ, BGISEQ; BGI], [Ion Torrent; Thermo Fisher]) have a history of accuracy and are supported by a wide range of analysis tools and bioinformatics tools and pipelines.^2,61^ Although there has been concern about lower accuracy in long-read sequencing platforms, error rates have fallen from 12–15% to 5% for ONT sequencers and 1% for SMRT compared to 0.1% for next-generation short-read sequencing.^1,2^ Despite ongoing technologic developments, there continues to be a need for practical tools to generate rapid and clinically relevant AMR data.

Short-read sequencing can be used for both whole-genome sequencing of bacterial pathogens, as well as the direct application to clinical samples for metagenomic sequencing, analysis, and detection of pathogens and AMR genes (ARGs).^54,66,132,162^ To date, there are no rapid library preparation protocols available for the generation of short-read sequencing libraries, which limits the feasibility of this technology for POC application.^116^ With increasingly rapid DNA extraction and library preparation techniques, the turnaround time (TAT) for short-read assembly-based approaches could be reduced.^133^ In contrast, rapid library preparation protocols do exist for long-read sequencing, and can be ready in as little as 15 min.^51^

Long-read sequencing platforms also have the advantage of detecting ARGs in reads that include complete genes along with flanking sequences that can be used to link the ARGs to bacterial taxa without the need for genome assembly.^59^ Although short-read data can be used for both assembly-based and read-based ARG detection,^158^ conservation of ARGs between species and on mobile genetic elements makes taxonomic assignment without prior assembly challenging.^10^ Genome assembly of short-read data for ARG detection linked to specific bacteria of interest requires higher genome coverage and more computing resources than read-based long-read ARG detection.^59^ As a result, detection of ARGs and respective taxonomic context from short-read data is time-consuming and suffers from a loss of information through quality control filtering and subsequent assembly of only a portion of the reads, resulting in the loss of some ARGs in the process. Long-read sequencing using ONT offers the speed and ease of computation with the ability for read-based detection of ARGs,^59^ as well as the ability to look at real-time sequence data, given that it creates data that are accessible during the run.^28,29,142^ In contrast, short-read sequence data are not available from platforms such as Illumina until after completion of the run.^67^ These features make long-read metagenomic sequencing an attractive option for rapid AMR detection to support stewardship, and are thus the focus of our scoping review.

As a newer technology, there is interest in comparing the application of metagenomic long-read sequencing for pathogen and ARG detection to established methods such as PCR, loop-mediated isothermal amplification (LAMP), and recombinase polymerase amplification (RPA) to detect specific target gene sequences by direct application to samples.^41,172^ We chose these other methods for our study as being either standard (PCR) or emerging tools (LAMP, RPA) in the rapid laboratory testing space. In 2009, the American Association for Clinical Chemistry published the minimum information requirements for reporting quantitative PCR (qPCR) tests (MIQE reporting guidelines) to ensure the relevance, accuracy, correct interpretation, and repeatability of qPCR experiments,^22^ as well as the minimum information requirements for reporting (meta)genomic studies (MIGS reporting guidelines).^47^ These guidelines provide a starting point for comparing different molecular methods for directly detecting respiratory pathogens and associated ARGs.

To our knowledge, there have been no published reviews that compare long-read metagenomic sequencing (directly applied to clinical samples) to PCR, LAMP, or RPA for detecting respiratory infections and ARGs in animals or humans. Specifically, our searches of the Ovid Medline/Embase, Cochrane Library, and Joanna Briggs Institute (JBI) Systematic Review Register on 2020 Dec 8, yielded no existing systematic or scoping review on this topic. Therefore, our objectives in this scoping review were to: 1) synthesize the knowledge about direct application of long-read metagenomic sequencing to clinical samples as an emerging technology for rapid identification of bacterial respiratory infections and related AMR; 2) compare long-read, direct metagenomic sequencing methods to other rapid laboratory tests of interest (i.e., qualitative/semi-quantitative/quantitative PCR, LAMP, and RPA) for nucleic acid detection; and 3) identify and characterize the methods applied in animal health settings with human health applications as comparators.

## Methods

### Protocol, search, and information sources

Our scoping review followed systematic methods outlined in the JBI Reviewer’s Manual^115^ and is reported according to the PRISMA Scoping Review (PRISMA-ScR) guidelines.^138^ A comprehensive strategy was developed in consultation with a librarian to identify articles that reported investigations of the rapid laboratory tests of interest (long-read metagenomic sequencing, PCR, LAMP, RPA), bacterial respiratory tract infections and/or pathogens, and AMR and/or ARGs in animals or humans (Table 1). The complete protocol (Suppl. Material) includes the complete search strings that we used to search MEDLINE, AGRICOLA, *BIOSIS Previews*, CABI, and Embase. The ONT website was searched for additional, applicable gray literature.^113^ We completed the first database and gray literature search on 2021 Jan 15, then updated them on 2022 Jan 19.

**Table 1.** Full electronic search string used to search MEDLINE via Ovid (https://ovidsp.ovid.com/) for articles about the laboratory tests of interest, bacterial respiratory infections, antimicrobial resistance, and turnaround time factors.

Search	Search statement
1	((genom* or metagenom* or amino acid or high throughput or base) adj3 sequence*).ti,ab.
2	exp *Amino acid sequence/ or exp *high throughput sequencing/ or exp *sequence analysis/ or exp *base sequence/ or exp DNA sequence/ or exp RNA sequence/
3	(rapid* or immediate* or "pen side" or "bed side" or "point of care" or "same day").ti. or exp "point of care testing"/
4	exp respiratory system/ or nose.mp. or noses.mp. or bronchia*.mp. or lung.mp. or lungs.mp. or breath.mp. or breathing.mp. or bronchial.mp. or bronchi.mp. or bronchioles.mp. or sinus.mp. or sinuses.mp. or pleura*.mp. or nasal.mp. or alveol*.mp. or trachea.mp. or respir*.mp. or vocal cord*.mp. or throat.mp.
5	(nanopore* or PacBio* or long-read* or MinION or PromethION or GridION or Voltrax).mp. [mp=title, abstract, original title, name of substance word, subject heading word, floating sub-heading word, keyword heading word, organism supplementary concept word, protocol supplementary concept word, rare disease supplementary concept word, unique identifier, synonyms]
6	exp polymerase chain reaction/ or Recombinant polymerase amplification/ or PCR.mp. or RPA.mp. [mp=title, abstract, original title, name of substance word, subject heading word, floating sub-heading word, keyword heading word, organism supplementary concept word, protocol supplementary concept word, rare disease supplementary concept word, unique identifier, synonyms]
7	(loop-mediated isothermal amplification or LAMP).mp. [mp=title, abstract, original title, name of substance word, subject heading word, floating sub-heading word, keyword heading word, organism supplementary concept word, protocol supplementary concept word, rare disease supplementary concept word, unique identifier, synonyms]
8	1 or 2 or 5 or 6 or 7
9	3 and 8
10	((resistan* and antibiotic*) or antimicrobial* or antimicrobial* or anti-bacterial* or antibacterial* or multidrug or multidrug or AMR or XDR or TDR or superbug* or superbug*).mp. [mp=title, abstract, original title, name of substance word, subject heading word, floating sub-heading word, keyword heading word, organism supplementary concept word, protocol supplementary concept word, rare disease supplementary concept word, unique identifier, synonyms]
11	4 or 10
12	9 and 11

### Eligibility criteria

We sought to include peer-reviewed published articles and relevant gray literature (e.g., white papers). Included studies had to report using long-read sequencing, PCR, LAMP, or RPA to detect nucleic acids, and the technique must have been applied directly to a respiratory sample (i.e., without prior culture and isolation) to detect bacterial respiratory pathogens, and/or bacterial ARGs. To date, only 2 methods dominate the long-read sequencing space: ONT and PacBio SMRT sequencing.^2^ We defined respiratory samples as those collected from the pulmonary system and the pleural space, including swabs, aspirates, washes, lavages, biopsies, or postmortem tissue samples. The samples could be in various states of storage or handling, such as fresh, frozen, spiked, or mock samples mixing host and pathogen DNA. We specifically searched for and considered “rapid” a term for real-time, same-day, or otherwise fast method that provided the required detection, identification, or confirmatory information within 48 h, but we allowed the term to evolve and be defined according to the context of the data extracted from included studies; it was not a screening criterion. No limits were applied to language, geographic location, or long-read sequencing types.

Articles were screened for eligibility via a 3-stage screening process by 2 independent reviewers (primary reviewer: O. Adewusi, secondary reviewers: Tianna Rusnak, Etienne De Jongh, Julia Grochowski). Article titles, abstracts, and keywords were screened in the first stage, and articles proceeded to secondary screening if both reviewers determined that all eligibility criteria were met or unclear (Suppl. Material). Secondary full-text screening by both reviewers included articles that met all eligibility criteria. We amended the protocol to add a third screening stage to apply a cutoff to articles published in or after 2009 to focus on technology advancements after the release of the MIQE and MIGS guidelines.^22,47^ Additionally, we limited article selection to those that reported test TAT and analytical sensitivity (limit of detection, LOD),^22^ aiming to describe the performance of the laboratory tests of interest. We excluded articles that did not report the application of these techniques for detecting nucleic acids through direct application to samples for bacterial respiratory infections or ARGs, that did not report TAT or LOD, or that were exclusively about bioinformatics tools, protocols, or pipelines. Review articles, conference abstracts, preprints, books, book chapters, theses, dissertations, commentaries, opinion pieces, editorials, and newspaper articles were excluded. The reasons for exclusion were documented. Reviewers resolved conflicts through discussion and had a third independent reviewer (S. Otto) available to resolve any conflicts if required.

### Data collection and synthesis

Search results were imported into EndNote (v.X9.2; Clarivate Analytics) for deduplication and then into Distiller SR (v.2.35; Evidence Partners) for additional deduplication, screening, and data extraction. We hand-searched reference lists of articles that passed second-level screening to identify any additional articles. Data extraction included authors, publication date, location of study, study design, objective, study population, sample size, participants’ health status, the type of sample collected, the laboratory technique used (and standard test for comparison), application of the analysis (experimental or laboratory assay), TAT, clinical and analytical sensitivity and specificity, and economic characteristics of the test. The last set of data extraction items focused on long-read sequencing methods (host depletion, pre-incubation, adaptive sequencing method, bait capture method, fragment length, the measure of quality, and intra- and inter-assay variations). One reviewer (O. Adewusi) extracted data in Distiller SR and analyzed it in Excel and Power BI (Microsoft). Extracted items were identified based on the MIQE^22^ and MIGS^47^ guidelines, as well as the knowledge needs of the study team. A narrative approach was used for data synthesis.

## Results

### Selection of information sources

**Figure 1.**
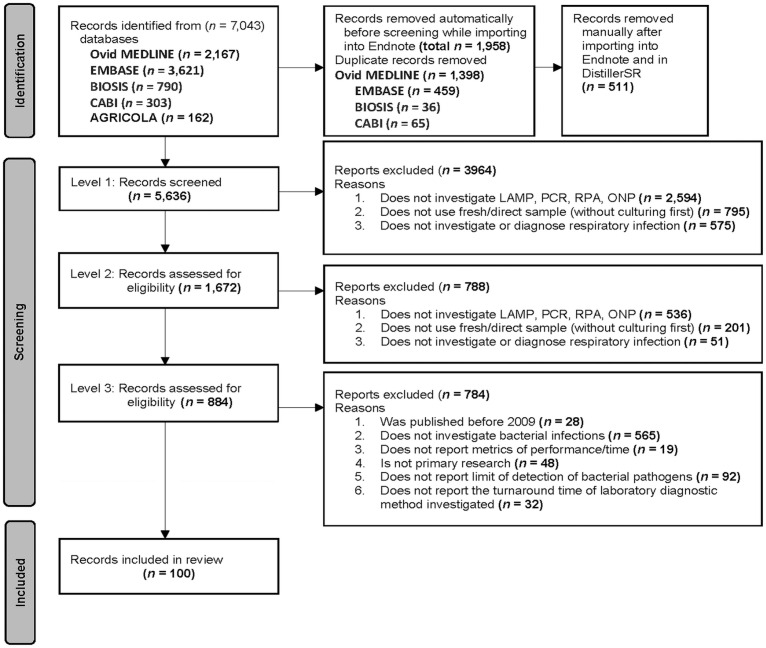
PRISMA scoping review flow diagram of the study selection process for the scoping review of the published literature on the direct application of laboratory tests to respiratory samples to detect bacterial pathogens and antimicrobial resistance.

The published and gray literature search identified 5,636 unique articles (Fig. 1). Primary, secondary, and tertiary screening excluded 3,964, 788, and 784 articles, respectively, leaving 100 articles that met the eligibility criteria for inclusion in our review. All included articles came from published literature; no gray literature articles met the inclusion criteria. There were no unresolved conflicts between the 2 reviewers for all screening levels.

### Characteristics of included studies

Studies investigating PCR were the most common (44 of 100), followed by LAMP (34 of 100), RPA (15 of 100) and long-read metagenomic sequencing (7 of 100, all 7 of which reported using ONT (Table 2; Suppl. Excel Table). Studies investigating human bacterial respiratory infections were the most common (81 of 100). Of the 19 studies investigating animal bacterial respiratory infections, 8 were on swine, and 5 were on cattle (1 feedlot, 2 dairy, and 2 both or unspecified; Table 3). The most common laboratory test reported in animal studies was LAMP, followed by RPA, PCR, and one ONT study. Studies published before 2014 only reported LAMP and PCR (Suppl. Excel Table). The first RPA study appeared in 2014, followed by the first ONT study in 2017. Most studies were conducted in Asia, Europe, and North America, with PCR and RPA being the most widespread methods. In contrast, relatively few studies were conducted in Africa, Australia, and South America (Suppl. Excel Table). Studies reporting ONT were limited to North America and Europe.

**Table 2.** Summary of studies by laboratory test method that report detection of bacterial pathogens ± antimicrobial resistance (AMR) genes via direct application to respiratory samples in humans and animals (*n* = 100).

Host	AMR gene detection	Method	No. of studies	List of citations
Human (*n* = 81)	Yes (*n* = 31)	ONT	4	^19,28,29,142^
		PCR	18	^26,27,46,48,50,58,62,72,82,97,101,107,117,137,139,143,150,160^
		LAMP	8	^42,63,84,86,103,104,108,173^
		RPA	1	^123^
	No (*n* = 50)	ONT	2	^6,57^
		PCR	23	^14,30,40,52,55,64,65,68,70,71,76,81,83,99,111,120,122,125,126,128,129,134,174^
		LAMP	17	^23,37,44,69,73,78,88,89,93,94,127,141,144,157,161,168,175^
		RPA	8	^17,98,102,121,151,155,156,167^
Animal (*n* = 19)	Yes (*n* = 1)	ONT	0	NA
		PCR	0	NA
		LAMP	0	NA
		RPA	1	^33^
	No (*n* = 18)	ONT	1	^16^
		PCR	3	^34,77,149^
		LAMP	9	^5,45,85,92,95,109,119,146,165^
		RPA	5	^60,90,145,169,170^

LAMP = loop-mediated isothermal amplification; NA = not applicable; ONT = Oxford Nanopore Technologies sequencing; RPA = recombinase polymerase amplification.

**Table 3.** Laboratory test methods by animal (*n* = 19) versus human (*n* = 81) host and by animal species reported by studies investigating the direct application to respiratory samples for detection of bacterial respiratory pathogens and antimicrobial resistance genes.

Test method	Animal	Human	Animal species (*n* = 19)	ONT	PCR	LAMP	RPA
ONT	1	6	Bovine (feedlot cattle)	—	—	—	1
PCR	3	41	Bovine (dairy cattle)	—	—	—	2
LAMP	9	25	Bovine (unspecified)	1	—	1	—
RPA	6	9	Caprine	—	—	—	1
Total	19	81	Equine	—	2	—	—
			Feline	—	—	1	—
			Ovine	—	—	—	2
			Swine	—	1	7	—

LAMP = loop-mediated isothermal amplification; ONT = Oxford Nanopore Technologies sequencing; RPA = recombinase polymerase amplification. Dash (—) indicates no study with this host species and assay combination.

### Bacterial respiratory pathogens and ARGs

Included articles identified 99 unique bacterial species (animal = 16; human = 83; Suppl. Excel Table). The animal bacterial pathogens and most common human bacterial pathogens (reported ≥3 times) are shown in Table 4. There was no overlap in reported bacterial species identified in human and animal studies with the exception of *B. bronchiseptica. Mycoplasma bovis* (*n* = 4) and *Streptococcus equi* (*n* = 3) were the most reported pathogens in animal studies, compared to *Staphylococcus aureus* (*n* = 26) and *Mycobacterium tuberculosis* (*n* = 24) in human studies. Thirty-two studies (human *n* = 31; animal *n* = 1) investigated ARG detection (Table 5; Suppl. Excel Table).

**Table 4.** Bacterial species identified as reported by studies on the direct application of laboratory tests to respiratory samples for detection of bacterial respiratory pathogens and antimicrobial resistance genes. All animal-reported bacterial species are included. For human studies, bacterial species reported in 3 or more studies are included.

Animal studies (*n* = 19)		Human studies (*n* = 81)	
Bacterial species	No. of studies	Bacterial species	No. of studies
*Mycoplasma bovis*	4	*Staphylococcus aureus*	26
*Streptococcus equi*	3	*Mycobacterium tuberculosis*	24
*Actinobacillus pleuropneumoniae*	2	*Streptococcus pneumoniae*	18
*Bordetella bronchiseptica*	2	*Klebsiella pneumoniae*	16
*Glaesserella* (*Haemophilus*) *parasuis*	2	*Pseudomonas aeruginosa*	14
*Mesomycoplasma* (*Mycoplasma*) *hyopneumoniae*	2	*Haemophilus influenzae*	13
*Pasteurella multocida*	2	*Escherichia coli*	12
*Chlamydia* (*Chlamydophila*) *felis*	1	*Mycoplasmoides* (*Mycoplasma*) *pneumoniae*	11
*Histophilus somni*	1	*Acinetobacter baumannii*	10
*Mannheimia haemolytica*	1	*Enterobacter cloacae*	9
*Mesomycoplasma* (*Mycoplasma*) *hyorhinis*	1	*Serratia marcescens*	7
*Mycoplasma capricolum*	1	*Stenotrophomonas maltophilia*	7
*Mycoplasma felis*	1	*Bordetella pertussis*	6
*Streptococcus equi* subsp. *zooepidemicus*	1	*Chlamydia pneumoniae*	6
		*Klebsiella oxytoca*	6
		*Moraxella catarrhalis*	6
		*Streptococcus pyogenes*	6
		*Legionella pneumophila*	5
		*Burkholderia cepacia*	4
		*Klebsiella aerogenes*	4
		*Proteus mirabilis*	4
		*Streptococcus agalactiae*	4
		*Acinetobacter calcoaceticus/baumannii* complex	3
		*Enterococcus faecalis*	3
		*Proteus* species	3

**Table 5.** Summary of studies reporting assays that detected multiple targets in the scoping review of direct application of laboratory tests to respiratory samples to detect bacterial pathogen species and antimicrobial resistance genes (ARGs).*

Host	Method	Detection in a single assay*				Detection in multiple assays†			Total
		>1 species + >1 ARG	>1 species + 1 ARG	>1 species + no ARGs	1 species + >1 ARG	1 species + 1 ARG	>1 species + no ARGs‡	No species + 1 ARG	
Human (*n* = 51)	ONT	1	—	2	3	—	—	—	6
	PCR	2	2	9	9	5	1	—	28
	LAMP	—	—	6	3	5	—	—	14
	RPA	—	—	2	—	1	—	—	3
Animal (*n* = 4)	PCR	—	—	1	—	—	—	—	1
	LAMP	—	—	1	—	—	—	—	1
	RPA	—	—	1	—	—	1	1	2

LAMP = loop-mediated isothermal amplification; ONT = Oxford Nanopore Technologies sequencing; RPA = recombinase polymerase amplification. Dash (—) indicates no study with this combination of detection of the number of bacterial species and ARGs.

* The study reported that multiple targets (bacterial species and/or ARGs) were detected within a single assay.

† The study reported the detection of multiple targets in multiple assays. There were no studies in which multiple assays detected one bacterial species or multiple bacterial species with multiple ARGs.

‡ The studies reported the detection of multiple bacterial targets, but no ARG targets, in multiple assays.

Fifty-three human studies reported single assays able to detect multiple bacterial pathogens, ARG targets, or various combinations of these directly from respiratory samples—ONT (*n* = 6), PCR (*n* = 28), LAMP (*n* = 15), and RPA (*n* = 4; Table 5, Suppl. Excel Table). Only one animal study reported multiplexing within a set of 11 RPA assays for different BRD pathogens and ARG targets: a) 1 conventional species-specific multiplex RPA assay targeting the 4 BRD pathogens (*Histophilus somni*, *M. bovis*, *Mannheimia haemolytica*, *Pasteurella multocida*); b) 2 species-specific real-time multiplex RPA assays targeting *M. haemolytica* and/or *M. bovis* and *P. multocida* and/or *H. somni*, respectively, with a novel competitive internal amplification control; c) 7 single assays, each with different ARG targets—*tet*(H), *tet*(R), *msr(*E), *mph(*E), *sul(*2), *flo(*R), and *erm(*42); and d) 1 targeting integrative and conjugative elements (ICE).^33^ One human study used droplet digital PCR, including 4 assays, each with 4 primer pairs for quantitative analysis of 16 different bacterial targets collectively.^174^

### Turnaround time

Ninety-seven studies reported test TATs with various definitions and inclusion of steps, of which 71 reported a TAT of 120 min or less; 52 of 71 described this as “rapid,” 12 of 97 as POC, 4 of 97 as both rapid and POC, and the remaining 3 as neither POC nor rapid (Suppl. Excel Table). Twenty studies reported TATs of 121–480 min (19 of 20 as rapid and 1 as neither POC nor rapid). Nine studies reported 481–2,880 min (all described as “rapid”).

The reported definition of TAT varied. Specific “sample-to-result” TAT (including sample preparation, DNA amplification [and library preparation for ONT] and detection) was reported in 44 of 97 studies (Table 6). There were patterns in the most commonly reported times—RPA, 30 min (3 of 6); LAMP, 60 min (4 of 9); and ONT, 360 min (2 of 3)—with PCR being more variable (Table 6). None of the ONT studies reported TATs <360 min. Of the remaining 56 studies, 36 reported that TAT included the DNA amplification and detection but not sample preparation, and 9 did not specify the processes in the reported TAT. The remaining 11 studies reported sample preparation only (*n* = 2), sample preparation and detection only (*n* = 1), sample preparation and DNA amplification only (*n* = 2), DNA amplification only (*n* = 3), and detection only (*n* = 3). Most studies investigating LAMP reported reaction time only (interpreted as DNA amplification and detection in this review).

**Table 6.** Times for studies that reported “sample-to-result” turnaround time (*n* = 44 of 97, including all steps from sample preparation through DNA amplification and detection) for the direct application of laboratory tests to respiratory samples to detect bacterial pathogens and antimicrobial resistance.

Reported time, min	ONT	PCR	LAMP	RPA
0–30*	—	—	—	3
31–60*	—	5	6	2
61–90*	—	4	3	—
91–120*	—	1	—	1
121–150	—	—	—	—
151–180	—	3	—	—
181–210	—	1	—	—
211–240	—	6	—	—
241–270	—	—	—	—
271–300	—	1	—	—
301–330	—	—	—	—
331–360	2	1	—	—
>360	1	4	—	—

LAMP = loop-mediated isothermal amplification; ONT = Oxford Nanopore Technologies sequencing; RPA = recombinase polymerase amplification. Dash (—) indicates no report for this assay and time combination.

* Reported turnaround times by test type that meet the World Health Organization (WHO) guidance for simple/rapid tests that provide results within 2 h.^152^ These represent the reports by test that fall within the WHO definition.

### Sample preparation

Our definition of sample preparation included decontamination of samples, nucleic acid extraction, and purification of obtained extracts for amplification or sequencing. Twenty studies reported using automated systems for sample preparation (Suppl. Excel Table), including 17 human studies and 3 animal studies (ONT, PCR, LAMP).^16,109,149^ In human settings, automated sample preparation was reported in 3 ONT, 9 PCR, and 5 LAMP studies.

### Sensitivity and specificity

The MIQE guidelines^22^ defined analytical sensitivity as the lowest concentration of analytes that can be detected within a reasonable or acceptable level of certainty when using an analytical tool. As well, the MIQE guidelines^22^ further noted that analytical sensitivity is typically expressed as the LOD, which is the concentration that can be detected with a reasonable level of certainty (with 95% probability being used commonly). Analytical specificity refers to the assay distinguishing between the desired target sequence and other targets and not detecting the undesired targets in the sample.^22^ Diagnostic or clinical sensitivity and specificity are the percentages of individuals with or without a given condition that the assay correctly identifies as positive or negative for that condition, respectively.^22^

#### Analytical sensitivity (LOD)

We identified 11 unique reported units of measurement in the 97 studies reporting numeric LOD values; 3 of the ONT studies did not report LOD but were included given our primary interest in this methodology (Table 7; Suppl. Excel Table). Thirty-five studies reported cfu: ONT (*n* = 1), PCR (*n* = 19), LAMP (*n* = 12), and RPA (*n* = 3). Thirty-one studies reported gene copies, DNA copies, genomes, or genome equivalents: ONT (*n* = 2), PCR (*n* = 14), and LAMP (*n* = 9). Other reported units included femtograms of DNA (12 of 97), picograms of DNA (9 of 97), and nanograms of DNA (1 of 97). Common denominators of the LOD attributes were milliliter (*n* = 41), microliter (*n* = 15), and reaction (*n* = 15). Others were swab (*n* = 1), sample (*n* = 1), test (*n* = 1), tube (*n* = 2), well (*n* = 1), LAMP zone (*n* = 1), and unspecified (*n* = 19). The most common unit of measurement for PCR (17 of 44) and LAMP (8 of 34) was cfu/mL; for RPA it was [gene] copy(ies)/reaction (7 of 15). The ONT LOD measures included cfu/mL, genomes/reaction, and genome equivalents/mL.

**Table 7.** Summary of studies reporting analytical and/or clinical sensitivity and/or specificity in the scoping review of direct application of laboratory tests to respiratory samples to detect bacterial pathogen species and antimicrobial resistance genes.

Measures reported	Analytical sensitivity		Analytical specificity		Clinical sensitivity		Clinical specificity	
	No	Yes	No	Yes	No	Yes	No	Yes
Animal	1	18	5	14	9	10	9	10
Human	2	79	35	46	25	56	26	55
Total (*n* = 100)	3	97	40	60	34	66	35	65

#### Analytical specificity

Of the 100 studies in our review, 60 (PCR = 24, LAMP = 22, and RPA = 14; human = 46, animal = 14) reported analytical specificity (Table 7; Suppl. Excel Table). These methods used known targets different from those of interest to determine analytical specificity. In contrast, none of the ONT studies reported analytical specificity. Studies often reported a qualitative, narrative description of these results. The most common word describing specificity was ‘negative,’ such as the samples from healthy controls, buffers used as controls, or blank controls, for non-targeted templates returning negative signals (e.g., amplification, fluorescence, or bands) when tested. “No cross-reactivity” or “no cross-reaction” were commonly used to describe analytical specificity.

#### Clinical sensitivity and specificity

Of the 66 (human = 46; animal = 20) studies reporting clinical sensitivity and specificity, PCR (*n* = 35) and LAMP (*n* = 20) had the highest numbers of articles compared to RPA (*n* = 8) and ONT (*n* = 3; Table 7). In 54 of the studies, results were presented as 2-way frequency tables to compare results with a putative gold standard assay. The methods of clinical sensitivity and specificity analysis were unspecified in 10 studies. The remaining 2 (1 ONT, 1 PCR) used a Bayesian latent class model^16^ and the Best estimate/La Place method^34^ for analysis, respectively.

### Costs of testing methodologies

Only 17 of 100 studies that we included in our review reported any information about the cost of the test (Suppl. Excel Table). Of this, one ONT study reported that reagents cost $270 USD per sample, not including any labor costs.^57^ The 2 LAMP studies only provided general comments about cost.^94,144^ Of the 2 RPA studies, 1 provided general comments about cost^167^; the other reported that the RPA per sample cost of their test was $11 USD compared to $2 USD for the comparable qPCR test.^171^ Although 12 PCR studies provided some information on cost, the variable inclusion of elements prevented a summary across studies. One study reported the cost of traditional PCR at $0.80 USD per sample^50^; another found a total cost of $8–10 USD.^17^

## Discussion

### Summary of evidence

We identified 100 studies reporting the direct application of long-read metagenomic sequencing, PCR, LAMP, or RPA to detect bacterial respiratory pathogens and ARGs in respiratory samples in animals and humans. The only long-read metagenomic sequencing method reported in papers included in the review was the ONT method. Overall, the included studies were human-centered, with only one animal study investigating ONT^16^ and only one animal study reporting ARG detection using RPA.^33^ The isothermal methods (LAMP, RPA) were used more frequently in animal studies than long-read sequencing and PCR. There was substantial heterogeneity in the reported LOD metrics and processes included in reporting total TAT linked to descriptions of test rapidity.

We found a considerable knowledge gap in animal studies, particularly those investigating ARGs across methods or those using long-read metagenomic sequencing methods for the direct detection of bacterial respiratory pathogens and AMR. The low number of papers that reported direct application of long-read methods highlights this opportunity for future research. An updated search on 2023 Feb 23, yielded 4 new publications that met our search criteria, acknowledging that the search records did not undergo screening by 2 reviewers. These studies reported using ONT,^31^ LAMP,^41^ and RPA (*n* = 2).^130,147^ A rapid, long-read metagenomic sequencing study reported using ONT to detect bacterial and fungal pathogens in human, ventilator-associated pneumonia cases compared to a combined standard of culture and qPCR for pathogen detection.^31^ They also compared metagenomic ARG detection to culture-based antimicrobial susceptibility testing and found high consistency between the ARGs detected from gram-negative bacterial pathogens with aminoglycoside and beta-lactam resistance phenotypes. Their median TAT for long-read metagenomic sequencing using ONT was 4.4 h. An additional long-read metagenomic sequencing paper published by our group^51^ did not meet the inclusion criteria because it did not report TAT. Our pilot study applied metagenomic ONT sequencing to deep nasopharyngeal swabs from chronically ill feedlot cattle to detect BRD pathogens and ARGs and compared the results to traditional bacterial culture and antimicrobial susceptibility testing.

These knowledge gaps present enormous opportunities to develop and further investigate the use of rapid laboratory tests for respiratory infections and AMR in animal health. To allow comparison, existing standardized metrics to determine the sample-to-result time and LOD may require further considerations. Comparability of tools will provide human and animal health practitioners with objective criteria to allow informed choices between rapid tests that best meet their needs to support antimicrobial stewardship through the identification of AMR. These criteria could include ease of deployment, adaptability to unique production settings, and the ability to generate easily interpretable results.

### Host, bacterial pathogens, and ARGs in animals and humans

We found that available research is highly human-centered, with very few species of animals considered, specifically cats,^109^ cattle,^5,16,33,169,170^ goats,^90^ horses,^34,149^ pigs,^45,77,85,92,95,119,146,165^ and sheep.^60,145^ One of the 99 bacterial pathogens reportedly identified, *B. bronchiseptica*, was the only organism reported in human and animal studies. *B. bronchiseptica* is a common respiratory pathogen of dogs and cats that was reported in cats^109^ and pigs,^165^ as well as in a study investigating biothreats and common respiratory pathogens in humans.^70^ The one study investigating AMR in animals focused on bacterial BRD pathogens and ARGs in feedlot cattle.^33^ The study reported the experimental transfer of ARGs through ICE from BRD pathogens to enteric pathogens.^33^ The human AMR studies were primarily focused on methicillin-resistant *S. aur-eus*^117,123,139,143,173^ and drug-resistant *M. tuberculosis*.^26,58^

### Application of the laboratory tests of interest in animals and humans

It was not a surprise that PCR was the most frequently reported assay given that it is a longer-standing technology than long-read metagenomic sequencing, LAMP, and RPA. However, starting in 2009, the isothermal methods (LAMP, RPA) were the most reported tests for direct application to respiratory samples in animal studies. Conversely, the metagenomic application of long-read sequencing for respiratory pathogens and ARGs is in a relatively early stage of development. The only animal ONT study included in our scoping review investigated *M. bovis* in pooled respiratory samples of cattle.^16^ The wide application of isothermal techniques for the direct detection of bacterial respiratory pathogens and ARGs in various human and animal settings could be because of the simplicity of operation, potential for a relatively short TAT, low LOD, and ease of interpretation of outputs (e.g., LAMP),^130,147,173^ which make these attractive methods to monitor and control outbreaks in resource-limited settings.^41^

We found that laboratory tests described as “rapid” had variable descriptions and inclusion of steps from sample collection to reporting of results. Importantly, there was variable reporting of the inclusion and time for DNA preparation before execution of the assay. Reported TATs ranged from a few minutes^19,102,104,144,145^ to 2 d,^83^ with no consistency or standardization on what constituted a description of rapid or POC or processes included in the reporting of TAT (e.g., sample-to-result, sample preparation, DNA amplification and analysis). This ambiguity remains despite the WHO definition of ‘simple/rapid’ laboratory tests that included specified test types (i.e., immunoassays), TAT range (10 min–2 h), adaptation to certain context or settings, equipment requirement, range of samples to be handled per time, and test storage requirement.^152^ The WHO definition^152^ does not allow for meaningful consideration or comparison of other methods such as long-read metagenomic sequencing because it does not clearly define what constitutes TAT and what steps should be included. Hence, the WHO definition is not a standard guideline for reporting TAT for all types of laboratory tests. The reported sample-to-result TATs of ONT, LAMP, and RPA were most commonly 6 h, 0.75 h, and 0.5 h, respectively. When reported TATs were ≤30 min, the process reported generally did not include sample and DNA preparation.^19,33,37,145,155^ There was high variability in the reported sample-to-result TAT of PCR. Other factors affecting sample-to-result time, such as settings, transportation, handling, and personnel, were not reported consistently. Variable reporting did not allow for a quantitative comparison between the tools. A more detailed description of the impact of TAT on evidence-based clinical management decisions was beyond the scope of our review but is an important area for future research.

Some examples of reported veterinary POC applications in our review were a LAMP-integrated microfluidic device and portable genetic analyzer for *Mycoplasma felis*, *B. bronchiseptica*, *Chlamydia* (*Chlamydophila*) *felis*, and feline herpesvirus 1 in cats,^109^ a RPA-lateral flow dipstick for rapid visual detection of *M. bovis* and *Mesomycoplasma* (*Mycoplasma*) *ovipneumoniae* in cattle and sheep,^60,145,170^ and a field application of RPA to detect *Mycoplasma capricolum* subsp. *capripneumoniae* in goats.^90^ The potential to optimize and develop accurate tools by seeking and targeting highly conserved pathogens or ARGs was also reported by some studies.^33,165,169^ The ability of PCR, LAMP, and RPA to be multiplexed for simultaneous detection of multiple organisms ±ARGs was highly variable. For polymicrobial samples, assays such as a single multiplex LAMP assay^109^ or multiple RPA assays (which may not be multiplex or real-time)^33^ could be required. However, there is concern about the loss of specificity in multiplexed LAMP assays.^8^ The limitation of multiplexed RPA is reduced amplification efficiency of one target compared to another because of competition among primer sets for recombinase proteins.^35^ Including a competitive internal amplification control in the assay (instead of additional primers specific for an internal control) can mitigate these detrimental interactions.^33^ However, this method requires post-amplification clean-up, visualization using gel electrophoresis, or use of exogenous synthetic DNA, all of which are not suitable for field application.^33^ This is in comparison to LAMP results, which can be read without a gel and further simplified by adding a lateral flow dipstick for visual interpretation.^8^ There were also various types and modifications of PCR to identify multiple targets directly from respiratory samples, such as a reverse-transcription PCR–electrospray ionization mass spectrometry,^70^ a DNA microarray assay,^99^ the BD MAX system,^122^ and the BioFire FilmArray pneumonia panel.^160^

The competitive displacement interactions in multiplex PCR, LAMP, and RPA assays depend on the concentration of DNA in the sample.^15^ Various tools can be used to quantify extracted DNA (e.g., fluorospectrometer). The animal ARG study included in our review relied on 11 different RPA assays, of which 2 were multiplexed.^33^ To ensure that each polymicrobial sample contained enough DNA for amplification and detection, the extracted DNA was normalized to 10 ng/μL, and then to a 50,000 genome copies/μL stock for each of the BRD pathogens. Compared to those high enough to induce a signal, targets with a lower concentrations may not do so, which could affect test specificity.^15^ The issue of DNA concentration could be avoided, in theory, for long-read methods as metagenomic sequencing continues despite low DNA concentration until sufficient genome coverage has been obtained, as reported in an investigation of human *M. tuberculosis* infections^142^; initial ARG detection was possible after 20 min of sequencing on a MinION outfitted with a R9.4 flow cell, provided the *M. tuberculosis* DNA concentrations were sufficient, with final results available within 150 min.^142^ A detailed review of the impacts of DNA concentration on direct application of these laboratory tests to respiratory samples is warranted and is another important area for future research.

When compared to PCR, LAMP, and RPA, a vital distinction inherent in long-read metagenomic sequencing is the ability to detect multiple organisms (cultivable and non-cultivable) and ARGs from a single sample in a single run. With long-read sequencing, reads of all the genetic material in the sample are generated,^2^ whereas targeted primers are required for PCR, LAMP, and RPA. The ability of long-read sequencing to identify and detect specific sequences within this milieu is based on the DNA quality, sequencing read length, coverage depth, and a bioinformatics pipeline that depletes host DNA and links the sequence data to databases of known organisms and ARGs.^21^ The long-read sequencing studies included in our review confirmed the importance of these factors for the performance of this metagenomic method.^16,19,29,57,142^ One study intended to investigate *M. bovis* for antimicrobial decision-making also identified additional *Mycoplasma* species (*Malacoplasma penetrans*, *Mycoplasma dispar*, *Mycoplasma wenyonii*, *Mycoplasmopsis arginini*, *Mycoplasmopsis bovirhinis*) in bronchoalveolar lavage samples from cattle.^16^ An optimized nanopore sequencing-based clinical metagenomics framework using the ONT ‘What’s in My Pot?’ pipeline to identify potential human respiratory pathogens and ARGs has been described.^29^ Sequencing identified ARGs for multiple antimicrobial classes, including aminoglycosides, cephalosporins, penicillins, other beta-lactams, fluoroquinolones, fusidane, sulfonamides, and trimethoprim. Within 5 min of sequencing, macrolide-resistance genes were detected in host-depleted samples and after 5 min in non–host-depleted samples. All ARGs were detected within 20 min of sequencing in host-depleted samples and 2 h of sequencing in non–host-depleted samples.^29^ In an adaptation of the ONT “genomic typing neighbour,” Prophyle (a metagenomic classifier) was successfully optimized to identify resistance-associated sequence elements and predicted macrolide and tetracycline resistance in human sputum metagenomic samples within 5 min of sequencing.^19^

### Analytical sensitivity and specificity

Of the included studies, all but 3 ONT studies reported numeric values of LOD. The LOD units included bacteria,^34,55^ cfu,^14,29,127,156^ genes/copies/genome equivalents,^6,37,170,174^ cumulative criterion unit,^90^ femtograms,^40,77,98^ nanograms,^95^ picograms,^120,121,161^ and organisms.^71^ Although these units are highly variable, they align with the MIQE recommendation that LOD can be expressed as relevant to the experiment.^22^ Some studies reported 2 units of LOD, calculating both femtograms and picograms of DNA and the genome equivalents,^50,60,70^ number of bacilli,^82^ or copies of DNA per reaction.^85,155^ Although the focus of our study was not numerical comparison of LOD data, future research may leverage this information for an in-depth analysis of different LODs. There were differences in LOD when different primer pair types were used for the same sample^17^ and for different organisms in a polymicrobial sample.^76,109,151^

Analytical specificity, on the other hand, was often reported as a qualitative, narrative description of the results of experiments in which the assay was applied to targets different from the target of interest. Assessment of analytical specificity for ONT sequencing is done through bioinformatics and is dependent on the organism and ARG sequences reported and being present in the reference databases.^59^ The sequence data generated from a sample can be used to identify bacterial taxa and ARGs with various levels of confidence based on the sequencing coverage depth and read length, the computational method used to compare samples data to reference sequences, and the scope and quality of the reference database.^21^ These factors are important considerations for inclusion in future guidelines.

### Considerations

The focus of our review was the detection of nucleic acids from bacterial respiratory pathogens and ARGs using the direct application of long-read metagenomic sequencing or other methods of interest (i.e., PCR, LAMP, or RPA) to respiratory samples. Studies focusing on clinical efficiency (e.g., patient wait time, health outcomes) rather than laboratory test efficiency were not considered. Also, studies focusing exclusively on bioinformatics tools and protocols and/or pipelines were excluded and are an important area for future review. We acknowledge that there are other potential test platforms in this space, such as the Luminex immunoassays, which were outside the scope of our review. This precluded a formal comparison of long-read sequencing to Luminex because these studies were not included. A review of U.S. Food and Drug Administration–approved tests for the detection of ARGs or AMR mutations for microorganisms identified Luminex platforms for use in detecting ARGs in human gram-positive and -negative bacterial isolates from blood, without any examples of respiratory application.^7^ Respiratory studies on Luminex seemed largely focused on viral pathogens,^79,136,154^ and thus were outside the scope of our review.

Most studies included in our review did not report on costs associated with the testing methodologies. Those that did were inconsistent, ranging from general comments that did not include dollar values to specific costs that varied from an overall estimate of cost per sample to a reagent cost per sample. The one ONT study reported substantially higher reagent costs per sample^57^ than the handful of RPA^171^ and PCR tests that reported specific numbers. This is not a surprise given the differences in how metagenomic, long-read sequencing methods are applied to samples compared to PCR, LAMP, and RPA. One must also consider that the cost of metagenomic sequencing is difficult to summarize given that its variability depends on sample type, amount of host DNA, and subsequent requirements for sequencing coverage to detect both pathogens and ARGs. Various metrics can be used to assess test costs, such as per-reaction, per-test, per-specimen, or per-patient costs. The variation in measurement units complicates direct comparisons across diverse testing platforms if costs are even included, underscoring the importance of using consistent metrics for accurate evaluations.

Next-generation sequencing technology has continued to evolve since the time of our last search for this review. Although outside of the scope of our review protocol, which was designed in late 2020, we conducted a focused search on 2023 Feb 23, for any study reporting the direct application of metagenomic short-read sequencing methods to detect respiratory pathogens and ARGs that reported TAT and LOD. We found 2 studies that reported direct application of metagenomic sequencing to human sputum, but no studies in the animal space. One study reported that this application gave genotypic ARG predictions in 44 h (Illumina MiSeq) and 16 h (Illumina MiniSeq).^142^ The other study combined the Illumina NextSeq platform with an automated library preparation device and internal-index adaptors to achieve early assignment of reads by first sequencing the barcodes, which enabled accurate pathogen identification in 9.1–10.1 h.^164^ This improved sample-to-result TAT and metagenomic application of short-read sequencing is a reflection of technology advancement and should be a focus for future review. We acknowledge that there are additional short-read metagenomic sequencing studies that demonstrate the ability to identify ARGs that did not meet our review criteria (e.g., direct application to respiratory sample to detect bacterial and ARG nucleic acids and reporting of TAT and LOD).^54,105,159^ Specifically, there are additional short-read and metagenomic sequencing studies on the respiratory microbiome and BRD pathogens of feedlot cattle that apply these methods for bacterial species and ARG detection.^3,25,43,54,75,112,163^

Limitations of short-read metagenomic sequencing methods for rapid pathogen and ARG detection still exist (e.g., lack of rapid library preparation and the need for genome assembly to assign ARGs to bacterial taxa).^59,140^ The increasing ability of long-read metagenomic sequencing methods to overcome these challenges make it an attractive option in the rapid metagenomic sequencing space.^51,59^ Although direct comparisons are rare, a recent study of human gut microbiota found that metagenomic sequencing using the ONT platform had superior ability to detect ARGs compared to short-read methods when using specimens with low numbers of bacteria.^106^

### Limitations

The rigorous and systematic approach for our study aimed to capture all eligible articles for review, but any scoping review runs the risk of articles not being included in the search strategy.^118^ We focused on the direct application of long-read metagenomic sequencing and other rapid laboratory methods of interest that eliminate the need for culture and isolation for rapid detection of bacterial respiratory infections and related AMR. Other rapid methods, such as immunoassays (e.g., Luminex) and isothermal methods other than LAMP and RPA, were not included in the scope of our review because they were not the focus of our work. We also focused on bacterial pathogens given the central question of ARG detection. The role of viruses in the pathogenesis of respiratory infections is documented and a vital area of future research.^38,166^ The same may also apply to parasites.^13^ The MIQE is a standard for reporting quantitative PCR results, and analytical sensitivity is one of the items on this list.^22^ Applying the qPCR MIQE guidelines to qualitative and/or semi-quantitative and/or quantitative PCR, LAMP, and RPA was necessary given the lack of similar reporting guidelines for other PCR applications and isothermal amplification assays. Further, the WHO statement for simple and rapid tests^152^ is not a standard guideline for reporting test TAT. The development of a robust guideline to accommodate the collation of different components of sample-to-result TAT for laboratory tools is an important area for future research and field implementation and comparison.

Test throughput is another important consideration when evaluating the efficiency of a laboratory test. Comparison of throughput was not a primary objective of our review, and there were very few instances where included studies directly reported on this aspect. The inconsistent definition and reporting of TAT compounded this issue. One included study reported on the comparison of LAMP to high-throughput next-generation sequencing for bacterial lower respiratory tract infections in humans, concluding that LAMP could be a promising option for swift and dependable clinical testing.^127^ A recent review suggested that isothermal methods provide potential for high-throughput screening assays^110^; however, assessment of throughput was lacking from all of the ONT studies included in our review, negating any formal comparison. This is another important area for future research.

## Conclusion

Our review highlights the urgent need for research on the direct application of long-read metagenomic sequencing methods to respiratory samples for pathogen and ARG detection in animals. Although there is extensive research on AMR in animal respiratory pathogens,^9,11,74,91^ our review identified a lack of studies on the direct application of laboratory tests to simultaneously detect bacterial pathogens and ARGs to inform clinical decisions. Our review highlights a paucity of data and literature for rapid laboratory tests to support global antimicrobial stewardship in animal health. In particular, there is a demonstrated international need to address the WHO^153^ recommendation for laboratory testing to support AMU in food animals as a pillar in future AMR strategies^56^ to leverage available technology and develop innovative laboratory tests. Given rapid technology advancements in this field, our review should be considered with emerging findings to make a comprehensive informed decision about future research.

The most critical advantage of long-read metagenomic sequencing over other methods included in our review is the ability to detect a wide range of targets from multiple samples of sequencing compared to PCR, RPA, and LAMP, which rely on a limited number of known pathogen and ARG targets and have limited multiplexing ability within a single assay. Long-read metagenomic sequencing methods have huge potential for ARG detection in multiple organisms within a single assay in that they can sequence all genomic material in the sample and identify organisms and ARGs by querying existing sequence databases and are not restricted to a limited number of predefined target sequences. Laboratory tests provide evidence to support treatment decisions by human and animal health practitioners. However, urgent and ongoing research is needed to close the knowledge gap for developing tests that provide more timely and accurate results for food animal livestock.^135^ Long-read sequencing technology is still very limited in use for direct, metagenomic application to detect respiratory bacterial pathogens and ARGs in animals and humans, but is a rapidly developing field.

## Supplemental Material

sj-pdf-1-vdi-10.1177_10406387241235968 – Supplemental material for Laboratory tools for the direct detection of bacterial respiratory infections and antimicrobial resistance: a scoping review Supplemental material, sj-pdf-1-vdi-10.1177_10406387241235968 for Laboratory tools for the direct detection of bacterial respiratory infections and antimicrobial resistance: a scoping review by Olufunto O. Adewusi, Cheryl L. Waldner, Patrick C. Hanington, Janet E. Hill, Claire N. Freeman and Simon J. G. Otto in Journal of Veterinary Diagnostic Investigation

sj-xlsx-2-vdi-10.1177_10406387241235968 – Supplemental material for Laboratory tools for the direct detection of bacterial respiratory infections and antimicrobial resistance: a scoping review Supplemental material, sj-xlsx-2-vdi-10.1177_10406387241235968 for Laboratory tools for the direct detection of bacterial respiratory infections and antimicrobial resistance: a scoping review by Olufunto O. Adewusi, Cheryl L. Waldner, Patrick C. Hanington, Janet E. Hill, Claire N. Freeman and Simon J. G. Otto in Journal of Veterinary Diagnostic Investigation
