# Supplementary material for: Laboratory tools for the direct detection of bacterial respiratory infections and antimicrobial resistance: a scoping review
Source: J Vet Diagn Invest. 2024 Mar 8;36(3):400–17. doi: 10.1177/10406387241235968 (PMC11110769; doi:10.1177/10406387241235968)
Supplement: sj-pdf-1-vdi-10.1177_10406387241235968 – Supplemental material for Laboratory tools for the direct detection of bacterial respiratory infections and antimicrobial resistance: a scoping review [file sj-pdf-1-vdi-10.1177_10406387241235968.pdf]

JVDI supplemental material

Adewusi OO, et al. Laboratory tools for the direct detection of bacterial respiratory pathogens and antimicrobial resistance: a scoping review

Supplementary Table 1. Completed reporting Items for Systematic reviews and Meta-Analyses extension for Scoping Reviews (PRISMA-ScR) Checklist.

Note: the reported pages/lines align with the word file that includes but does not show tracked changes in the revised manuscript (i.e., it is in "Simple Markup" view).

SECTION	ITEM	PRISMA-ScR CHECKLIST ITEM	REPORTED ON PAGE #
**TITLE**			
Title	1	Identify the report as a scoping review.	Page 1
**ABSTRACT**			
Structured summary	2	Provide a structured summary that includes (as applicable): background, objectives, eligibility criteria, sources of evidence, charting methods, results, and conclusions that relate to the review questions and objectives.	Page 2 (note – the summary is unstructured per the journal guidelines)
**INTRODUCTION**			
Rationale	3	Describe the rationale for the review in the context of what is already known. Explain why the review questions/objectives lend themselves to a scoping review approach.	Pages 4-6 (Lines 42-121)
Objectives	4	Provide an explicit statement of the questions and objectives being addressed with reference to their key elements (e.g., population or participants, concepts, and context) or other relevant key elements used to conceptualize the review questions and/or objectives.	Page 6 (Lines 121-128)
**METHODS**			
Protocol and registration	5	Indicate whether a review protocol exists; state if and where it can be accessed (e.g., a Web address); and if available, provide registration information, including the registration number.	Pages 6-7 (Lines 131-133) and S1 Protocol.
Eligibility criteria	6	Specify characteristics of the sources of evidence used as eligibility criteria (e.g., years considered, language, and publication status), and provide a rationale.	Page 7-8 (145-159) and S1 Protocol
Information sources*	7	Describe all information sources in the search (e.g., databases with dates of coverage and contact with authors to identify additional sources), as well as the date the most recent search was executed.	Page 7 (Lines 137-143)
Search	8	Present the full electronic search strategy for at least 1 database, including any limits used, such that it could be repeated.	Page 7 (Lines 133-143) and Table 1, S1 Protocol
Selection of sources of evidence†	9	State the process for selecting sources of evidence (i.e., screening and eligibility) included in the scoping review.	Page 8 (Lines 160-175)
Data charting process‡	10	Describe the methods of charting data from the included sources of evidence (e.g., calibrated forms or forms that have been tested by the team before their use, and whether data charting was done independently or in duplicate) and any processes for obtaining and confirming data from investigators.	Pages 8-9 (Lines 177-192)

SECTION	ITEM	PRISMA-ScR CHECKLIST ITEM	REPORTED ON PAGE #
Data items	11	List and define all variables for which data were sought and any assumptions and simplifications made.	Pages 8-9 (Lines 177-192)
Critical appraisal of individual sources of evidence§	12	If done, provide a rationale for conducting a critical appraisal of included sources of evidence; describe the methods used and how this information was used in any data synthesis (if appropriate).	N/A
Synthesis of results	13	Describe the methods of handling and summarizing the data that were charted.	Pages 8-9 (Lines 177-192)
**RESULTS**			
Selection of sources of evidence	14	Give numbers of sources of evidence screened, assessed for eligibility, and included in the review, with reasons for exclusions at each stage, ideally using a flow diagram.	Page 9 (Lines 195-200, Figure 1)
Characteristics of sources of evidence	15	For each source of evidence, present characteristics for which data were charted and provide the citations.	Pages 9-10, Lines 202-215, Table 2, Supplementary Table S2
Critical appraisal within sources of evidence	16	If done, present data on critical appraisal of included sources of evidence (see item 12).	N/A
Results of individual sources of evidence	17	For each included source of evidence, present the relevant data that were charted that relate to the review questions and objectives.	Pages 10-15, Lines 202-320, Tables 2-7, and Supplementary Table S2
Synthesis of results	18	Summarize and/or present the charting results as they relate to the review questions and objectives.	Pages 10-15, Lines 202-320, Tables 2-7, and Supplementary Table S2
**DISCUSSION**			
Summary of evidence	19	Summarize the main results (including an overview of concepts, themes, and types of evidence available), link to the review questions and objectives, and consider the relevance to key groups.	Pages 15-25 (Lines 322-547)
Limitations	20	Discuss the limitations of the scoping review process.	Pages 25-26 (Lines 549-598)
Conclusions	21	Provide a general interpretation of the results with respect to the review questions and objectives, as well as potential implications and/or next steps.	Pages 26-27 (Lines 579-604)
**FUNDING**			
Funding	22	Describe sources of funding for the included sources of evidence, as well as sources of funding for the scoping review. Describe the role of the funders of the scoping review.	Page 28 (Lines 617-621)

JBI = Joanna Briggs Institute; PRISMA-ScR = Preferred Reporting Items for Systematic reviews and Meta-Analyses extension for Scoping Reviews.

* Where *sources of evidence* (see second footnote) are compiled from, such as bibliographic databases, social media platforms, and Web sites.

† A more inclusive/heterogeneous term used to account for the different types of evidence or data sources (e.g., quantitative and/or qualitative research, expert opinion, and policy documents) that may be eligible in a scoping review as opposed to only studies. This is not to be confused with *information sources* (see first footnote).

‡ The frameworks by Arksey and O'Malley (6) and Levac and colleagues (7) and the JBI guidance (4, 5) refer to the process of data extraction in a scoping review as data charting.
§ The process of systematically examining research evidence to assess its validity, results, and relevance before using it to inform a decision. This term is used for items 12 and 19 instead of "risk of bias" (which is more applicable to systematic reviews of interventions) to include and acknowledge the various sources of evidence that may be used in a scoping review (e.g., quantitative and/or qualitative research, expert opinion, and policy document).

From: Tricco AC, Lillie E, Zarin W, O'Brien KK, Colquhoun H, Levac D, et al. PRISMA Extension for Scoping Reviews (PRISMAScR): Checklist and Explanation. Ann Intern Med. 2018;169:467–473. doi: 10.7326/M18-0850.

S1 Scoping Review Protocol: When we need answers fast: A scoping review of long-read sequencing and other rapid methods for the diagnosis of respiratory infections

Authors

*Olufunto O. Adewusi[1], Cheryl L. Waldner[2], Janet E. Hill[3], Amreen Barbujee[1], Patrick Hannington[4], Simon J. G. Otto[1,5]

*Corresponding author: Funto Adewusi
Email: olufunto@ualberta.ca

Author affiliations
[1] HEAT-AMR Research Group, University of Alberta, School of Public Health
[2] University of Saskatchewan, Western College of Veterinary Medicine, Department of Large Animal Clinical Sciences
[3] University of Saskatchewan, Western College of Veterinary Medicine, Department of Veterinary Microbiology
[4] University of Alberta, School of Public Health
[5] Thematic Area Lead, Healthy Environments, Centre for Health Communities, School of Public Health, University of Alberta

1. Introduction

Antimicrobial resistance (AMR) is a global health crisis [1]. In 2015, the World Health Organization developed a Global Action Plan for AMR urging every member state to adopt and adapt five objectives to their national context [1]. The WHO, FAO and OIE made joint commitments to improve antimicrobial stewardship in the human and animal sectors, including investment in new diagnostic tools [2]. Evidence-based veterinary medicine based on diagnostic testing promotes antimicrobial stewardship in livestock management [2].

In Canada, bovine respiratory disease (BRD) management is the most common reason for parenteral antimicrobial use in feedlot cattle [3]. Antimicrobial use (AMU) protocols for B[4]RD are typically guided by clinical risk assessment using demographic information (source, age, weight class, breed), temperature measurements, and behaviour monitoring to make metaphylaxis and treatment decisions [5]. The BRD complex includes a variety of bacterial and viral pathogens - *Mannheimia haemolytica*, *Mycoplasma bovis*, *Pasteurella multocida*, *Histophilus somni*, bovine viral diarrhea virus (BVDV), bovine respiratory syncytial virus (BRSV), bovine herpesvirus 1 (BoHV-1), and parainfluenza three virus (PI3V) [6]. Laboratory-based diagnostic testing for bacterial BRD pathogens in live animals

currently relies on culture-based diagnostic methods for bacteria and antibiotic susceptibility testing [7]. These phenotypic tests take 5-7 days to receive results [8] and may be missing some bacteria due to differential growth rates and demanding requirements for laboratory cultivation [8]. As a result, improved rapid diagnostic tests are needed to provide more timely and accurate information for veterinarians to manage BRD in feedlot cattle. The changing international environment may require diagnostic information to justify AMU in livestock in the near future [4].

Rapid and immediate pen side identification of pathogens and AMR determinants to guide antimicrobial selection in individual animals is not yet an option. However, methods to provide diagnostic information within 1-2 days would be a vast improvement to inform evidence-based AMU at pen or herd management group [9]. Laboratory diagnostic methods that may provide rapid sample-to-result are long-read sequencing technology, polymerase chain reaction (PCR) [10], recombinase polymerase amplification (RPA), and loop-mediated isothermal amplification (LAMP) of target/specific gene sequences.

The PCR, LAMP and RPA methods are limited to known target sequences [11]. The polymerase chain reaction is a popular technology for detecting low-abundance nucleic acids to identify pathogens and AMR genes [12]. However, pathogen identification requires a known, specific gene target, as does AMR gene detection. The process requires multiple cycles of heating for DNA denaturing, specific probe-directed amplification, and synthesis, followed by detection of the target using a thermocycler [13], electrophoresis, or in real-time using either fluorogenic probes [14] or intercalating agents [15]. If multiplexed, it can identify more than one target sequence in a single assay but still has limits to the number of target sequences. In contrast, isothermal amplification techniques such as LAMP and RPA occur at one reaction temperature under simple conditions (e.g., water bath), eliminating the limitation of thermocycling in PCR, making point-of-care applications more realistic [16]. The LAMP method uses a set of four target-specific primers to recognize particular sites flanking the target DNA sequence, and amplification occurs at a constant temperature of 60 – 65°C [16]. Depending on detection chemistry and instrumentation, LAMP products can be detected as early as one hour. While RPA takes a bit longer (30-90 minutes), it can be run at a lower temperature (37-42 ^{o}C). Importantly, PCR, LAMP and RPA methods can all be applied directly to samples, precluding the need for isolation of organisms. This greatly reduces the time to receive diagnostic results, which is crucial for a diagnostic strategy for BRD management.

Second and third generation sequencing methods can also be applied directly to metagenomic samples[17], [18]. Short-read methods are more efficient for sequencing genomes of isolated organisms, which is not timely in rapid testing. Conversely, long-read sequencing techniques, such as The Oxford Nanopore platform (MinION, GridION, and PromethION), provide great promise for rapid diagnostic testing through direct metagenomic application to respiratory samples [19]. The development of

VolTRAX library preparation offers the potential to deploy mobile long-read sequencing in non-laboratory environments [20]. In addition, long-read techniques for metagenomic sequencing can discover novel sequences since the method does not rely on the amplification of target sequences. This opportunity for rapid sequencing of known and unknown genes will be helpful to inform treatment and management protocols for infectious diseases. Therefore, it is essential to summarize what is currently known about these rapid diagnostic technologies to inform the future development of a BRD laboratory diagnostic strategy.

In this scoping review, we will summarize the current state of knowledge regarding the direct application of long-read metagenomic sequencing technology to respiratory samples to diagnose respiratory infections in animals and humans. We will collect emerging insights concerning the methods, opportunities, and barriers for long-read sequencing of bacteria, viruses, virulence genes, and AMR genes. Further, we will compare long-read sequencing with other direct rapid molecular technologies for gene identification and characterization, specifically PCR, LAMP and RPA.

During a preliminary search of Ovid Medline/Embase, Cochrane Library and Joanna Briggs Institute Systematic Review Register on December 8, 2020 (see Appendix for preliminary search string), we found no existing scoping review or systematic reviews on this topic. The search indicated that relevant research exists for review.

2.1 Rationale

Rapid and efficient diagnostic testing strategies are required to meet future requirements to support AMU in livestock. Current methods take up to 5-7 days and rely on traditional culture and antimicrobial susceptibility testing. Rapid methods such as long-read metagenomics sequencing, PCR, RPA, or LAMP will support antimicrobial stewardship by providing information about known bacterial and viral pathogens, virulence genes, and AMR genes [16]. Exploring these new opportunities for rapid diagnostic testing will support the development of new diagnostic methods for BRD management and antimicrobial stewardship efforts. [21]. Short-read sequencing techniques are not well suited for direct application to respiratory samples and thus excluded from this review. However, PCR, LAMP and RPA can be valuable tools for the rapid identification and characterization of known target genes (specific for pathogen identification, antimicrobial resistance or virulence).

2.2 Research question

What is the current state of knowledge regarding rapid diagnostic tools for respiratory infections and related antimicrobial resistance in humans and animals?

Objectives:

1. To synthesize available knowledge in peer-reviewed literature and relevant grey literature (e.g., white papers) about the direct sample application of long-read sequencing for rapid diagnosis of respiratory infections and related antimicrobial resistance.
2. To compare long-read, direct sequencing methods to other rapid diagnostic techniques for nucleic acid detection.

2.3 Method

2.3.1 Inclusion criteria

Types of participants

Any study that includes animals and humans of any age with respiratory infections diagnosed using long-read sequencing, RPA, LAMP or PCR will be included. Any study that uses long-read sequencing, PCR, LAMP, or RPA to detect, identify, diagnose or confirm the presence of respiratory infections in humans or animals will be included.

Concept

Studies must describe the use of rapid technology and diagnosis of respiratory infection as primary points of interest. The study must include at least one of the techniques of interest: long-read sequencing, RPA, LAMP or PCR. There are currently only two methods available for long-read sequencing, also known as third-generation sequencing: nanopore methods from Oxford Nanopore Technology (ONT) and PacBio's single-molecule real-time sequencing (SMRT).

The comparator groups are other rapid diagnostic technologies such as PCR, LAMP and RPA. Rapid refers to a real-time, same-day or otherwise fast method that provides the required detection, identification, diagnostic or confirmatory information within 48 hours. Our outcome of interest is the diagnosis, detection, identification or confirmation of bacterial or viral respiratory infections, AMR genes and related, integrative and conjugative elements (ICEs) and plasmids, and/or virulence factors in samples from the respiratory tract. Also, we will allow the term 'rapid' to evolve and be defined according to the context of the final data set.

Context

There were no limits applied to language, geographical location, long-read sequencing types and date published.

Types of studies

The review will include published peer-reviewed articles. We will also hand-search the websites of technology companies to capture relevant proprietary primary research (e.g., White Papers). We would

also hand-search references and citations of most relevant articles (that is, articles that make it through second-level screening).

2.3.2 Exclusion Criteria

- Preprints, books, book chapters, theses, dissertations, commentary, opinion pages, editorials, newspaper articles
- Describes research that does not investigate the use of long-read sequencing or other pertinent methods (e.g., PCR, LAMP or RPA).
- Describes research that does not investigate rapid methods (i.e., results within 48 hours).
- Describes research that is not for specific detection of nucleic acids (i.e., it is research on antigen detection).
- Describes research that does not investigate the diagnosis, detection, confirmation, or identification of respiratory infections, pathogens or AMR genes and their related ICEs or plasmids, and/or virulence factors for respiratory pathogens.
- Research focusing on bioinformatics tools and protocols/pipelines.

2.3.3 Approach

The scoping review will follow the framework outlined in the Joanna Briggs Institute Reviewer's Manual, which includes identifying the research question, finding relevant studies, selecting studies, charting the data, and reporting the results [22]. The reporting guidelines developed by Tricco et al. in their extension of the PRISMA checklist for scoping reviews will be followed [23]. The process of selecting relevant inclusion or exclusion criteria variables required frequent team discussions. The iterative process will continue as we design relevant data extraction templates and variables of interest.

Search strategy

We will develop search strategies in consultation with a librarian and execute the search in the following databases: MEDLINE®, AGRICOLA™, *BIOSIS Previews*®, CABI and EMBASE®. We will hand-search the websites of technology companies to capture relevant proprietary primary research. We will review reference lists of included articles and future articles citing articles to identify any articles missed by the search. Outlined below are the main/target content areas and the proposed search terms in each domain. An initial search was completed in Ovid (Medline and Embase) using a preliminary search string; informal analysis of the terms used in the titles, abstracts and indexes of relevant articles identified

other terms pertinent to the search string. Synonyms and variations on the main terms are included in the more comprehensive search string.

The final search string (see Appendix) will be adapted and applied across the above databases. The reference lists from the included studies will be reviewed to identify additional articles of relevance using the snowball technique.

Table 1: Search term blocks for based on research question

Terms	Proposed search terms
Rapid diagnosis	Direct application of rapid and/or point-of-care diagnostic methods to samples to detect nucleic acids
Laboratory methods	a. Long-read sequencing
	b. Other rapid methods (PCR, RPA, LAMP)
respiratory tract infections +/- AMR	Respiratory tract infections in humans and animals that may/may not include information about AMR

2.3.4 Study selection and Screening Process

Two independent reviewers will screen articles at all levels of screening per the descriptions below for each level. When disagreements arise concerning the inclusion of a particular reference, the reviewers will discuss until they reach a consensus. In a situation where they cannot reach an agreement, then a third researcher will arbitrate. A third level of screening was added on Nov 2, 2021 to focus the review on questions of specific interest to the project team after obtaining a large number (>500) of articles for data extraction after level 2 screening (see Appendix 2). Furthermore to the third level screening, we made a decision to add a level 3b screening starting on January 24, 2022 to focus the review on one question (rather than multiple) of special interest to the project team after obtaining 271 articles for data extraction following level 3a screening (see Appendix 3).

Level 1 Screening

- Two independent reviewers will screen the full text of each article.. They will review disagreements to determine if the screening questions are working as intended. If required, screening questions will be modified and a protocol amendment dated. Once this is complete, each reviewer will review half of the remaining articles independently for efficiency.
- We will use a stacked questionnaire to screen the titles and abstracts captured by the initial search by two independent reviewers.

- If the article fully or partially meets the inclusion criteria (i.e., all screening questions are answered either 'yes' or 'unsure'), the paper will proceed to Level 2 screening.
- If the article meets any one of the exclusion criteria, it will not proceed to Level 2 screening, and the reason(s) for exclusion will be indicated.

Level 2 Screening:

- Two independent reviewers will screen the full text of each article.
- In Level 2 screening, only articles that meet all the inclusion criteria will be included in the review. 'Unsure' is not an option at this stage.
- One or more answers of "No" or "Unsure" to the Level 2 screening questions below lead to the exclusion of the article; information regarding the reason(s) for exclusion will be recorded.
- Google Translate™ will be used to screen non-English full article text.

Level 3 screening

Level 3 screening for the 594 full articles initially at the data extraction stage (appendix 2)

- Include only papers that focus on the diagnosis or investigation of bacterial diseases.
- Include all papers that report laboratory diagnostic qualitative and quantitative metrics of interest

Level 3b screening

Level 3b screening for 271 full articles initially at data extraction stage

- Include only papers that report limit of detection (aka detection limit, analytical sensitivity.)
- Include all papers that report assay turnaround time (aka run-time, hands-on-time)

Screening Questions

Level 1: the following questions will be answered using a stacked form to screen each title and abstract:

If the answer to any of the above screening questions is "no," the article will not move forward to secondary screening. An answer of "unsure" warrants additional investigation and the article will move forward to secondary screening.

For screening articles retrieved in 2021	For screening articles retrieved in 2022
1. Does the research involve investigating a long-read sequencing method or other method of interest (i.e., RPA, LAMP or PCR) specifically to detect nucleic acids?<br>*Note: diagnostic method (variable) of interest must be the focus of the study, the selection should be based on the focus of the study (must be included in the goal or objectives statement), methods (data must have been collected/extracted on the variable of interest), and/or results (results on the variable of interest must have been presented)*	1. Is this a review, commentary, a research note (that is, not a primary research?)
2. Does the research focus on direct application of the technique to a sample? (i.e., the sample is not cultured first.)	2. Does the research involve investigation of bacterial respiratory infections, pathogens or AMR genes, ICEs or plasmids associated with respiratory pathogens or respiratory samples
3. Does the research focus on respiratory infections, pathogens, AMR or virulence genes associated with respiratory pathogens or respiratory samples?	3. Does the research involve investigating a long-read sequencing method or other method of interest (i.e., RPA, LAMP or PCR) specifically to detect nucleic acids?<br>*Note: diagnostic method (variable) of interest must be the focus of the study, the selection should be based on the focus of the study (must be included in the goal or objectives statement), methods (data must have been collected/extracted on the variable of interest), and/or results (results on the variable of interest must have been presented)*
4. Should this abstract proceed to the next level?	4. Does the research focus on direct application of the technique to a sample? (i.e., the sample is not cultured first.)
	5. Should this abstract proceed to the next level?

Level 2: The following questions guide the screening of each full article:

For screening articles retrieved in 2021	For screening articles retrieved in 2022
1. Does the research involve investigating a long-read sequencing method or other methods of interest (i.e., RPA, LAMP or PCR) specifically to detect nucleic acids?<br>***Note:*** *diagnostic method (variable) of interest must be the focus of the study, the selection should be based on the focus of the study (must be included in the goal or objectives statement), methods (data must have been collected/extracted on the variable of interest), and/or results (results on the variable of interest must have been presented)*	1. Is this a review, commentary, a research note (that is, not a primary research?)
2. Does the research focus on the direct application of the technique to a sample? (i.e., the sample is not cultured first.)	2. Does the research involve investigation of bacterial respiratory infections, pathogens or AMR genes, ICEs or plasmids associated with respiratory pathogens or respiratory samples
3. Does the research focus on respiratory infections, pathogens or AMR or virulence genes associated with respiratory pathogens or respiratory samples?	3. Does the research involve investigating a long-read sequencing method or other method of interest (i.e., RPA, LAMP or PCR) specifically to detect nucleic acids?<br>*Note: diagnostic method (variable) of interest must be the focus of the study, the selection should be based on the focus of the study (must be included in the goal or objectives statement), methods (data must have been collected/extracted on the variable of interest), and/or results (results on the variable of interest must have been presented)*
4. Should this article proceed to the next level?	4. Does the research focus on direct application of the technique to a sample? (i.e., the sample is not cultured first.)
5.	5. Does this article report on the limit of detection, assay turnaround time or error rate (for nanopore sequencing papers)?<br>a. Limit of detection or detection limit or analytical sensitivity<br>b. Run-time (synonyms: Turn-around time; hands-on time)<br>c. Estimated error rate, base error rate (not considered for non-nanopore sequencing papers)
	6. Should this article proceed to the next level?

Level 3 Screening questions (2 reviewers)

For efficiency in the screening process, the articles retrieved in 2022 would have gone through this stage in level 2.

For screening articles retrieved in 2021	For screening articles retrieved in 2022
**1.** Does this study focus on the diagnosis or investigation of bacterial infections?	
**2.** Does this study report **at least one** of the following pieces of information numerically (one of these terms or a reasonable synonym)? If yes to any one of these, then include the paper for data extraction.<br>a. Run-time (synonyms: Turn-around time; hands-on time)<br>b. Sensitivity and/or specificity (analytical or diagnostic/clinical/epidemiological); estimated error rate, base error rate (synonyms: precision, accuracy)<br>c. Limit of detection or limit of quantification<br>d. Amplification efficiency.	

We will assess the agreement between the inclusion decisions of the two reviewers using the kappa statistic measure of agreement [24].

Data extraction

EndNote X9 and Distiller will be used to manage the citations. All eligible articles will be uploaded to EndNote X9 for automatic and manual removal of duplicates. The remaining articles will be uploaded to DistillerSR® and checked again for duplicates. Eligibility for inclusion will be determined with the software-created screening forms.

A data extraction form will be created in DistillerSR®. Data extracted will summarize the metadata, population, intervention, comparators and outcome of the articles.

Characteristics of the study, including:

- Year of publication
- Type of document
- Country: the setting of study participants, as reported by the author (e.g., animal, human, clinic, farm, laboratory-based)
- Year(s) of data collection
- Host species
- Sample type: nasal swab, nasopharyngeal swab, bronchoalveolar lavage, post-mortem respiratory tissue samples
- Sample host and characteristics
    - Human (Age, sex, health or risk status etc.)
    - Animals (healthy vs sick; exposure status etc.)
    - Laboratory-stored samples (frozen, fixed, etc.)
- Synthetic/contrived/mock samples

Type of laboratory diagnostic tool used

- Long-read sequencing, LAMP, RPA, PCR

Description of and results for factor(s) investigated, including:

- Run-time (hands-on time, turn-around time)
- Measures of quality, as adapted from the minimum requirement for (meta)genome and qPCR publication (annex 2)
- Measures of precision and accuracy - Sensitivity and specificity (diagnostic, clinical, epidemiologic)

2.3.5 Presentation of Results

We will present the results in a narrative summary format with the aid of tables and figures as required. Each important result of interest will have a narrative summary. Tables will include characteristics of the studies, comparisons based on the factors investigated.

REVISED
11:23 am, Feb 10, 2022

References

1. Organization, T.W.H., *Global Action Plan on Antimicrobial Resistance.* Official records of the Sixty-eighth World Health Assembly 2015. **WHA68/2015/REC/1**: p. 28.
2. Organisation, W.H., *Global Framework for Development & Stewardship to Combat Antimicrobial Resistance.* WHO/EMP/IAU/2017.08 2017.
3. Brault, S.A., et al., *Antimicrobial Use on 36 Beef Feedlots in Western Canada: 2008-2012.* Front Vet Sci, 2019. **6**: p. 329.
4. Aidara-Kane, A., et al., *World Health Organization (WHO) guidelines on use of medically important antimicrobials in food-producing animals.* Antimicrob Resist Infect Control, 2018. **7**: p. 7.
5. Noffsinger, T., K. Lukasiewicz, and L. Hyder, *Feedlot Processing and Arrival Cattle Management.* Vet Clin North Am Food Anim Pract, 2015. **31**(3): p. 323-40, v.
6. Klima, C.L., et al., *Pathogens of bovine respiratory disease in North American feedlots conferring multidrug resistance via integrative conjugative elements.* J Clin Microbiol, 2014. **52**(2): p. 438-48.
7. Steeve Giguere, J.F.P.a.P.M.D., *Chapter 7: Antimicrobial stewardship in Animals*, in *Antimicrobial therapy in Veterinary medicine* 2013. p. 117 - 132.
8. Pardon, B. and S. Buczinski, *Bovine Respiratory Disease Diagnosis: What Progress Has Been Made in Infectious Diagnosis?* Vet Clin North Am Food Anim Pract, 2020. **36**(2): p. 425-444.
9. D'Costa, V.M., et al., *Antibiotic resistance is ancient.* Nature, 2011. **477**(7365): p. 457-461.
10. Mirza, H., et al., *A Rapid, High-Throughput Viability Assay for Blastocystis spp. Reveals Metronidazole Resistance and Extensive Subtype-Dependent Variations in Drug Susceptibilities.* Antimicrobial Agents and Chemotherapy, 2011. **55**(2): p. 637-648.
11. Garibyan, L. and N. Avashia, *Polymerase Chain Reaction.* Journal of Investigative Dermatology, 2013. **133**(3): p. 1-4.
12. Pal, A. and A.K. Chakravarty, *Advanced genomic techniques for studying immune-response genes*, in *Genetics and Breeding for Disease Resistance of Livestock*. 2020. p. 209-234.
13. Institute, N.H.G., *Polymerase Chain Reaction (PCR) Fact Sheet.* Website, 2020.
14. Su, W., et al., *Microfluidic platform towards point-of-care diagnostics in infectious diseases.* J Chromatogr A, 2015. **1377**: p. 13-26.
15. Patterson, A.S., et al., *Electrochemical real-time nucleic acid amplification: towards point-of-care quantification of pathogens.* Trends Biotechnol, 2013. **31**(12): p. 704-12.
16. Zhao, Y., et al., *Isothermal Amplification of Nucleic Acids.* Chemical Reviews, 2015. **115**(22): p. 12491-12545.
17. Hause, B.M., et al., *An inactivated influenza D virus vaccine partially protects cattle from respiratory disease caused by homologous challenge.* Vet Microbiol, 2017. **199**: p. 47-53.
18. Roberts, R.J., M.O. Carneiro, and M.C. Schatz, *The advantages of SMRT sequencing.* Genome Biology, 2013. **14**(6): p. 405.
19. Amarasinghe, S.L., et al., *Opportunities and challenges in long-read sequencing data analysis.* Genome Biol, 2020. **21**(1): p. 30.
20. Jain, M., et al., *Nanopore sequencing and assembly of a human genome with ultra-long reads.* Nature Biotechnology, 2018. **36**(4): p. 338-345.
21. Technologies, O.N., *Large insights into microorganisms.* White paper, 2018: p. 24.
22. Manual, J.R.s., *Chapter 10: Assessing and presenting results.*
23. Tricco AC, L.E., Zarin W, O'Brien KK, Colquhoun H, Levac D, et al. , *PRISMA Extension for Scoping Reviews (PRISMA-ScR): Checklist and Explanation.* Ann Intern Med. , 2018. **16**(1): p. 1-10.
24. Orwin, R.G., *Evaluating coding decisions*, in *The handbook of research synthesis.*, Russell Sage Foundation: New York, NY, US. p. 139-162.

Appendix 1

	Ovid MEDLINE(R) ALL <1946 to December 24, 2020>	
#	**Search Statement**	**Results**
1	((genom* or metagenom* or amino acid or high throughput or base) adj3 sequence*).ti,ab.	172468
2	exp *Amino acid sequence/ or exp *high throughput sequencing/ or exp *sequence analysis/ or exp *base sequence/ or exp DNA sequence/ or exp RNA sequence/	765516
3	(rapid* or immediate* or "pen side" or "bed side" or "point of care" or "same day").ti. or exp "point of care testing"/	163558
4	exp respiratory system/ or nose.mp. or noses.mp. or bronchia*.mp. or lung.mp. or lungs.mp. or breath.mp. or breathing.mp. or bronchial.mp. or bronchi.mp. or bronchioles.mp. or sinus.mp. or sinuses.mp. or pleura*.mp. or nasal.mp. or alveol*.mp. or trachea.mp. or respir*.mp. or vocal cord*.mp. or throat.mp.	2029200
5	(nanopore* or PacBio* or long-read* or MinION or PromethION or GridION or Voltrax).mp. [mp=title, abstract, original title, name of substance word, subject heading word, floating sub-heading word, keyword heading word, organism supplementary concept word, protocol supplementary concept word, rare disease supplementary concept word, unique identifier, synonyms]	10837
6	exp polymerase chain reaction/ or Recombinant polymerase amplification/ or PCR.mp. or RPA.mp. [mp=title, abstract, original title, name of substance word, subject heading word, floating sub-heading word, keyword heading word, organism supplementary concept word, protocol supplementary concept word, rare disease supplementary concept word, unique identifier, synonyms]	770933
7	(loop-mediated isothermal amplification or LAMP).mp. [mp=title, abstract, original title, name of substance word, subject heading word, floating sub-heading word, keyword heading word, organism supplementary concept word, protocol supplementary concept word, rare disease supplementary concept word, unique identifier, synonyms]	21691
8	1 or 2 or 5 or 6 or 7	1497547
9	3 and 8	16829
10	((resistan* and antibiotic*) or antimicrobial* or antimicrobial* or anti-bacterial* or antibacterial* or multidrug or multidrug or AMR or XDR or TDR or superbug* or superbug*).mp. [mp=title, abstract, original title, name of substance word, subject heading word, floating sub-heading word, keyword heading word, organism supplementary concept word, protocol supplementary concept word, rare disease supplementary concept word, unique identifier, synonyms]	591046
11	4 or 10	2563842
12	9 and 11	**2167**
	After automatic deduplication in Endnote	**769**

	Ovid MEDLINE(R) ALL <1946 to January 18, 2022>	
#	**Search Statement**	**Results**
1	((genom* or metagenom* or amino acid or high throughput or base) adj3 sequence*).ti,ab.	364963
2	exp *Amino acid sequence/ or exp *high throughput sequencing/ or exp *sequence analysis/ or exp *base sequence/ or exp DNA sequence/ or exp RNA sequence/	1204196

3	(rapid* or immediate* or "pen side" or "bed side" or "point of care" or "same day").ti. or exp "point of care testing"/	382242
4	exp respiratory system/ or nose.mp. or noses.mp. or bronchia*.mp. or lung.mp. or lungs.mp. or breath.mp. or breathing.mp. or bronchial.mp. or bronchi.mp. or bronchioles.mp. or sinus.mp. or sinuses.mp. or pleura*.mp. or nasal.mp. or alveol*.mp. or trachea.mp. or respir*.mp. or vocal cord*.mp. or throat.mp.	5460287
5	(nanopore* or PacBio* or long-read* or MinION or GridION or PromethION or Voltrax).mp. [mp=ti, ab, ot, nm, hw, fx, kf, ox, px, rx, ui, sy, tn, dm, mf, dv, dq]	28197
6	exp polymerase chain reaction/ or Recombinant polymerase amplification/ or PCR.mp. or RPA.mp. [mp=ti, ab, ot, nm, hw, fx, kf, ox, px, rx, ui, sy, tn, dm, mf, dv, dq]	2121062
7	(loop-mediated isothermal amplification or LAMP).mp. [mp=ti, ab, ot, nm, hw, fx, kf, ox, px, rx, ui, sy, tn, dm, mf, dv, dq]	66824
8	1 or 2 or 5 or 6 or 7	3354737
9	3 and 8	39639
10	((resistan* and antibiotic*) or antimicrobial* or anti-microbial* or anti-bacterial* or antibacterial* or multidrug or multidrug or AMR or XDR or TDR or superbug* or superbug*).mp. [mp=ti, ab, ot, nm, hw, fx, kf, ox, px, rx, ui, sy, tn, dm, mf, dv, dq]	1301638
11	4 or 10	6616925
12	9 and 11	7205
13	limit 12 to yr="2021 -Current"	1382
14	**After deduplication**	**1033**

	**Embase <1974 to 2020 December 24>**	
**#**	**Search Statement**	**Results**
1	((genom* or metagenom* or amino acid or high throughput or base) adj3 sequence*).ti,ab.	175807
2	exp *Amino acid sequence/ or exp *high throughput sequencing/ or exp *sequence analysis/ or exp *base sequence/ or exp DNA sequence/ or exp RNA sequence/	392705
3	(rapid* or immediate* or "pen side" or "bed side" or "point of care" or "same day").ti. or exp "point of care testing"/	192447
4	exp respiratory system/ or nose.mp. or noses.mp. or bronchia*.mp. or lung.mp. or lungs.mp. or breath.mp. or breathing.mp. or bronchial.mp. or bronchi.mp. or bronchioles.mp. or sinus.mp. or sinuses.mp. or pleura*.mp. or nasal.mp. or alveol*.mp. or trachea.mp. or respir*.mp. or vocal cord*.mp. or throat.mp.	3086923
5	(nanopore* or PacBio* or long-read* or MinION or PromethION or GridION or Voltrax).mp. [mp=title, abstract, heading word, drug trade name, original title, device manufacturer, drug manufacturer, device trade name, keyword, floating subheading word, candidate term word]	11294
6	exp polymerase chain reaction/ or Recombinant polymerase amplification/ or PCR.mp. or RPA.mp. [mp=title, abstract, heading word, drug trade name,	1195052

	original title, device manufacturer, drug manufacturer, device trade name, keyword, floating subheading word, candidate term word]	
7	(loop-mediated isothermal amplification or LAMP).mp. [mp=title, abstract, heading word, drug trade name, original title, device manufacturer, drug manufacturer, device trade name, keyword, floating subheading word, candidate term word]	38622
8	1 or 2 or 5 or 6 or 7	1643286
9	3 and 8	19230
10	((resistan* and antibiotic*) or antimicrobial* or antimicrobial* or anti-bacterial* or antibacterial* or multidrug or multidrug or AMR or XDR or TDR or superbug* or superbug*).mp. [mp=title, abstract, heading word, drug trade name, original title, device manufacturer, drug manufacturer, device trade name, keyword, floating subheading word, candidate term word]	613570
11	4 or 10	3623245
12	9 and 11	**3621**
	**After automatic deduplication in Endnote**	**3162**

	Embase <1974 to 2022 January 18>	
**#**	**Search Statement**	**Results**
1	((genom* or metagenom* or amino acid or high throughput or base) adj3 sequence*).ti,ab.	182407
2	exp *Amino acid sequence/ or exp *high throughput sequencing/ or exp *sequence analysis/ or exp *base sequence/ or exp DNA sequence/ or exp RNA sequence/	411114
3	(rapid* or immediate* or "pen side" or "bed side" or "point of care" or "same day").ti. or exp "point of care testing"/	205809
4	exp respiratory system/ or nose.mp. or noses.mp. or bronchia*.mp. or lung.mp. or lungs.mp. or breath.mp. or breathing.mp. or bronchial.mp. or bronchi.mp. or bronchioles.mp. or sinus.mp. or sinuses.mp. or pleura*.mp. or nasal.mp. or alveol*.mp. or trachea.mp. or respir*.mp. or vocal cord*.mp. or throat.mp.	3320043
5	(nanopore* or PacBio* or long-read* or MinION or GridION or PromethION or Voltrax).mp. [mp=title, abstract, heading word, drug trade name, original title, device manufacturer, drug manufacturer, device trade name, keyword heading word, floating subheading word, candidate term word]	14358
6	exp polymerase chain reaction/ or Recombinant polymerase amplification/ or PCR.mp. or RPA.mp. [mp=title, abstract, heading word, drug trade name, original title, device manufacturer, drug manufacturer, device trade name, keyword heading word, floating subheading word, candidate term word]	1304330
7	(loop-mediated isothermal amplification or LAMP).mp. [mp=title, abstract, heading word, drug trade name, original title, device manufacturer, drug manufacturer, device trade name, keyword heading word, floating subheading word, candidate term word]	42977
8	1 or 2 or 5 or 6 or 7	1775172
9	3 and 8	21236
10	((resistan* and antibiotic*) or antimicrobial* or anti-microbial* or anti-bacterial* or antibacterial* or multidrug or multidrug or AMR or XDR or TDR or superbug* or superbug*).mp. [mp=title, abstract, heading word, drug trade name, original title, device manufacturer, drug manufacturer, device trade name, keyword heading word, floating subheading word, candidate term word]	661094
11	4 or 10	3896802

12	9 and 11	4637
13	limit 12 to yr="2021 - 2023"	934
	After deduplication	256

	**Results**	**BIOSIS (Search output December 24, 2020)**
		**Search statment**
	**73**	**After automatic deduplication in Endnote**
	**133**	**Rerun January 19, 2022**
	**766**	**After automatic deduplication in Endnote**
# 9	790	#8 AND #1<br>*Indexes=BIOSIS Previews Timespan=All years*
# 8	23,163	#7 AND #4<br>*Indexes=BIOSIS Previews Timespan=All years*
# 7	398,156	#6 OR #5<br>*Indexes=BIOSIS Previews Timespan=All years*
# 6	7,018	TS=(Long-read sequenc*) OR (TS=(nanopore* or PacBio* or long-read* or GridION or MinION or PromethION or Voltrax))<br>*Indexes=BIOSIS Previews Timespan=All years*
# 5	393,238	(TI=(polymerase chain reaction OR PCR OR LAMP OR loop-mediated isothermal amplification OR RPA OR recombinant polymerase amplification)) OR (TI=(amino acid sequences OR DNA amplification OR RNA amplification OR nucleotide sequences)) or (TI=(genom* OR metagenom* OR "amino acid" OR "high throughput" OR "base sequence*"))<br>*Indexes=BIOSIS Previews Timespan=All years*
# 4	2,324,709	#3 OR #2<br>*Indexes=BIOSIS Previews Timespan=All years*
# 3	2,174,252	TS=(respiratory system or nose or noses or bronchial or lung or lungs or breath or breathing or bronchial or bronchi or bronchioles or sinus or sinuses or pleura or nasal or alveolar or trachea or respiration or vocal cord or throat)<br>*Indexes=BIOSIS Previews Timespan=All years*
# 2	169,950	(TS= (((resistan*) AND (antibiotic* OR antimicrobial* OR antimicrobial* OR anti-bacterial* OR antibacterial* OR multidrug OR “multi*drug”)) OR AMR OR XDR OR TDR OR “super*bug*” OR superbug*)) *AND* **LANGUAGE:** (English) *AND* **DOCUMENT TYPES:** (Article)<br>*Indexes=BIOSIS Previews Timespan=All years*
# 1	157,127	(TI=(rapid* OR immediat* OR "pen side" OR "bedside" OR "bed side" OR "point of care" OR "same day"))<br>*Indexes=BIOSIS Previews Timespan=All years*

	Results	CABI (Search output December 24, 2020)
		**Search statement**
	**40**	**After automatic deduplication in Endnote**
	**66**	**Rerun January 19, 2022**
	**238**	**After automatic deduplication in Endnote**
# 10	**303**	#9 AND #7 *Indexes=CAB Abstracts Timespan=All years*
# 9	366,816	#8 OR #3 *Indexes=CAB Abstracts Timespan=All years*
# 8	278,336	TS=(respiratory system or nose or noses or bronchial or lung or lungs or breath or breathing or bronchial or bronchi or bronchioles or sinus or sinuses or pleura or nasal or alveolar or trachea or respiration or vocal cord or throat) *Indexes=CAB Abstracts Timespan=All years*
# 7	7,150	#6 AND #1 *Indexes=CAB Abstracts Timespan=All years*
# 6	584,476	#5 OR #4 OR #2 *Indexes=CAB Abstracts Timespan=All years*
# 5	14,195	(TS= (loop-mediated isothermal amplification or LAMP) OR TS=(recombinase polymerase amplification or RPA)) *Indexes=CAB Abstracts Timespan=All years*
# 4	1,187	TS=(Long-read sequenc*) OR TS=(nanopore* or PacBio* or long-read* or MinION or PromethION or GridION or Voltrax) *Indexes=CAB Abstracts Timespan=All years*
# 3	94,142	TS= (((resistan*) AND (antibiotic* OR antimicrobial* OR antimicrobial* OR anti-bacterial* OR antibacterial* OR multidrug OR “multi*drug”)) OR AMR OR XDR OR TDR OR “super*bug*” OR superbug*) *Indexes=CAB Abstracts Timespan=All years*
# 2	572,225	(de=(polymerase chain reaction or PCR) or de=(amino acid sequences or dna amplification or rna amplification or gene mapping or molecular genetics techniques or nucleotide sequences) or ts=(genom* or metagenom* or "amino acid" or "high throughput" or "base sequence*")) *Indexes=CAB Abstracts Timespan=All years*
# 1	48,661	TI=(rapid* OR immediat* OR "pen side" OR "bedside" OR "bed side" OR "point of care" OR "same day") *Indexes=CAB Abstracts Timespan=All years*

Result	**AGRICOLA (search output of December 24, 2020)**
	**Search statement**
42	**Rerun January 19, 2022**
**162**	ti(rapid* OR immediat* OR "pen side" OR "bedside" OR "bed side" OR "point of care" OR "same day") AND ab(rapid* OR immediat* OR "pen side" OR "bedside" OR "bed side" OR "point of care" OR "same day") AND noft(genom* OR metagenom* OR "high throughput" OR "amino acid" OR "polymerase chain reaction*" OR "base sequence" OR "base sequences" OR "hybridization*" OR "nanopore technology*" OR "MinION*" OR PromethION OR GridION OR Voltrax OR "long read" OR PCR OR LAMP OR "isothermal loop mediated*" OR "recombinase polymerase*" OR RPA) AND noft(((respiratory OR nose OR noses OR pharynx OR pharyngeal OR nasal OR bronchial OR lung OR lungs OR breath OR breathing OR bronchial OR bronchi OR bronchioles OR sinus OR sinuses OR pleura OR nasal OR alveolar OR trachea OR respiration OR vocal cord OR throat) OR (resistan* OR antibiotic* OR antimicrobial* OR antimicrobial* OR anti-bacterial* OR antibacterial* OR multidrug OR "multi*drug" OR AMR OR XDR OR TDR OR "super*bug*" OR superbug*))) AND noft(respiratory OR nose OR noses OR pharynx OR pharyngeal OR nasal OR bronchial OR lung OR lungs OR breath OR breathing OR bronchial OR bronchi OR bronchioles OR sinus OR sinuses OR pleura OR nasal OR alveolar OR trachea OR respiration OR vocal cord OR throat)

Appendix 2

Genomic ASSETS scoping review: UPDATE on efficient data extraction

Introduction

This scoping review aims to describe current knowledge about direct application of long-read metagenomic sequencing technology to respiratory samples for the diagnosis of respiratory infections in animals and humans. In addition, we will compare the performance of long-read sequencing with other direct rapid molecular technologies for gene identification and characterization, specifically PCR, LAMP and RPA. Furthermore, we will collect emerging insights on how experts have used these molecular laboratory diagnostic tools to detect bacteria, viruses, virulence genes, and AMR genes directly from respiratory samples and evaluate their effectiveness based on cost, reliability and turn around time.

What we have done so far.

We executed the scoping review protocol designed to address the research question identified together with the Genomic ASSETS team. The School of Public Health librarian helped to optimize the search executed on January 15, 2021 to retrieve relevant studies from five databases – MEDLINE®, AGRICOLA™, *BIOSIS Previews*®, CABI and EMBASE®. Two reviewers screened over 5,000 titles and abstracts at the first level screening and then over 1,500 full articles at the second level.

With 594 full articles for data extraction, we need a strategy to refine the scope for data extraction of a subset of information from this large pool within the broader goal of this review. We have tagged these references to support a decision making process that targets data extraction for the purposes of Funto's thesis.

Tags

We created tags for each of the 594 in DistillerSR references to support the objectives of the study. The tags are

1. **Year of publication -** format – **yyyy**
2. **Type of laboratory diagnostic tool(s) - PCR, LAMP, RPA** and long-read metagenomic? sequencing).
3. Type of respiratory infection or focus of investigation **- (bacterial, viral and others (AMR, plasmids, ICE(s) MGEs, fungal, and virulence genes).**
4. Type of host or source of respiratory sample **- Humans and Animals.**

Result of tagging

Table 1: Number of references tagged human, animal, bacteria and others in Distiller

Lab diagnostic test	Total	Human	Animal	Bacteria	Virus	**Others
LAMP	105	80	25	39	61	5
Long read metagenomic sequencing	10	10	0	5	5	0
PCR	454	396	57	221	221	12
RPA	29	15	14	12	18	0
		501	96	277	305	17
		Human	Animal	Bacteria	Virus	Others
All lab diagnostic tests		501	96	277	305	17

Proposed action:

Conduct level 3 screening for the 594 full articles initially at the data extraction stage

1) Include only papers that focus on the diagnosis or investigation of bacterial diseases.
2) Include all papers that report laboratory diagnostic qualitative and quantitative metrics of interest

Level 3 Screening questions (2 reviewers)

3. Does this study focus on the diagnosis or investigation of bacterial infections?
4. Does this study report **at least one** of the following pieces of information numerically (one of these terms or a reasonable synonym)? If yes to any one of these, then include the paper for data extraction.
    a. Run-time (synonyms: Turn-around time; hands-on time)
    b. Sensitivity and/or specificity (analytical or diagnostic/clinical/epidemiological); estimated error rate, base error rate (synonyms: precision, accuracy)
    c. Limit of detection or limit of quantification
    d. Amplification efficiency.

Note: these items were identified as variables of interest to compare the performance of long-read metagenomic sequencing to RPA, LAMP, and/or PCR (1, 2).

Table *2*: Adapted minimum information *requirement* for publication[1, 2]

	Assay characteristics	Detail
**Sample**	Sample acquisition	Fresh (point-of-care) vs stored
	Sample handling	If stored, is the storage conditions described?
	Sample preparation	Nucleic acid extraction method
**Assay**		Target sequences/genome
	Library construction	Number of reads, Reads (length, mapped, raw)
		Reference database (for read preprocessing and consensus building)
	Sequencing method	Single/multiple (DNA polymerase; Recombinase protein)
**Assay performance**		Run-time; Turn-around time; hands-on time
		Analytical sensitivity, Analytical specificity<br>Analytical Specificity must validated empirically with direct experimental evidence (electrophoresis, melting profile, DNA sequencing, amplicon size, and/or restriction enzyme digestion)<br>Diagnostic sensitivity, Diagnostic specificity
		Intra-assay variation (repeatability) (Yes/No)
		When multiple targets are investigated
		Amplification efficiency
		Limit of detection; Limit of quantification
		Point-of-care (Yes/No);<br>Setting (limited resources or not)
**Assembly**	**Assembly**	Assembly method, estimated error rate, method of calculation, Base error rate/accuracy
**Finishing strategy**	**Status**	Complete or draft, Genome coverage/variations

References

1. FAIRsharing.org: MIxS – MIGS/MIMS; Minimum Information about a (Meta)Genome sequence. Retrieved on October 21, 2021 from
2. Stephen A Bustin, Vladimir Benes, Jeremy A Garson, Jan Hellemans, Jim Huggett, Mikael Kubista, Reinhold Mueller, Tania Nolan, Michael W Pfaffl, Gregory L Shipley, Jo Vandesompele, Carl T Wittwer, The MIQE Guidelines: *M*inimum *I*nformation for Publication of *Q*uantitative Real-Time PCR *E*xperiments, *Clinical Chemistry*, Volume 55, Issue 4, 1 April 2009, Pages 611–622

Appendix 3

Figure 1: Result of tagging 271 articles after level 3a screening

	Sensitivity	Time	Limit of detection (LOD)	Total	Total (2009 and beyond)
LAMP	23	12	21	56	41
PCR	118	46	29	193	136
RPA	7	3	7	17	12
	148	61	57		
